# Cynandione A Alleviates Neuropathic Pain Through α7-nAChR-Dependent IL-10/β-Endorphin Signaling Complexes

**DOI:** 10.3389/fphar.2020.614450

**Published:** 2021-01-27

**Authors:** Qiao-Qiao Han, Min Yin, Zi-Ying Wang, Hao Liu, Jun-Ping Ao, Yong-Xiang Wang

**Affiliations:** ^1^ King’s Lab, Shanghai Jiao Tong University School of Pharmacy, Shanghai, China; ^2^ Jiangsu Key Laboratory for the Research and Utilization of Plants Resources, Institute of Botany, Jiangsu Province and Chinese Academy of Sciences, Nanjing, China; ^3^ State Key Laboratory of Oncogenes and Related Genes, Shanghai Cancer Institute, Shanghai Jiao Tong University, Shanghai, China

**Keywords:** cynandione A, neuropathic pain, interleukin-10, β-endorphin, α7 nicotinic acetylcholine receptor, microglia

## Abstract

Cynandione A, an acetophenone isolated from Cynanchum *Wilfordii* Radix, exhibits antineuropathic pain effect. This study further explored the target molecule and signaling mechanisms underlying cynandione-A-induced antineuropathic pain. Intrathecal injection of cynandione A significantly attenuated mechanical allodynia in neuropathic rats and substantially increased spinal expression of IL-10 and β-endorphin but not dynorphin A. Cynandione A treatment also enhanced expression of IL-10 and β-endorphin but not α7 nicotinic acetylcholine receptors (nAChRs) in cultured microglia. The IL-10 antibody attenuated cynandione-A-induced spinal or microglial gene expression of β-endorphin and mechanical allodynia, whereas the β-endorphin antiserum blocked cynandione-A-induced mechanical antiallodynia but not spinal or microglial IL-10 gene expression. The α7 nAChR antagonist methyllycaconitine significantly reduced cynandione-A-induced mechanical antiallodynia and spinal or microglial expression of IL-10 and β-endorphin. Furthermore, cynandione A stimulated microglial phosphorylation of PKA, p38, and CREB in an α7-nAChR-dependent manner, and treatment with their inhibitors attenuated cynandione-A-induced mechanical antiallodynia and spinal or microglial expression of IL-10 and β-endorphin. In addition, cynandione A stimulated spinal phosphorylation of the transcription factor STAT3, which was inhibited by methyllycaconitine, the PKA activation inhibitor or IL-10 antibody. The STAT3 inhibitor NSC74859 also abolished cynandione-A-induced mechanical antiallodynia and spinal expression of β-endorphin. These findings suggest that cynandione A suppresses neuropathic pain through α7-nAChR-dependent IL-10/β-endorphin signaling pathway in spinal microglia.

## Introduction

Cynanchum *Wilfordii* has long been used in the East Asian countries especially in China, Korea, and Japan as a traditional herb medicine for the treatment of insomnia, anxiety, anemia, senescence, and various geriatric diseases (Gong et al., 1988; Hwang et al., 1999; Koo et al., 2015). As an acetophenone, cynandione A is the major active ingredient of Cynanchum *Wilfordii*. It has been demonstrated to reduce LPS-induced expression of TNF-α, IL-6, IL-1β, nitric oxide and prostaglandin E2 in BV-2 microglia, RAW264.7 macrophages and primary microglia, as well as in septic mice and neuropathic rats (Yang et al., 2014; Kim et al., 2015; Huang et al., 2017). It also attenuated glutamate-induced cytotoxicity and mitigated ischemic injuries in rats with cerebral ischemia (Yue et al., 2012) and protected cultured cortical neurons from toxicity induced by hydrogen peroxide, L-glutamate, and kainate (Lee et al., 2000). In addition, intrathecal injection of cynandione A in neuropathic rats markedly stimulated spinal β-endorphin expression and alleviated mechanical allodynia and thermal hyperalgesia, which were blocked by the microglial metabolic inhibitor minocycline, the β-endorphin antibody and μ-opioid receptor antagonist. These results suggest that cynandione A alleviates neuropathic pain through upregulation of spinal microglial expression of β-endorphin (Huang et al., 2017). However, little is known about the upstream signaling and target molecule mechanisms of cynandione A, although its antinociception was blocked by the α7 nicotinic acetylcholine receptor (α7 nAChR) antagonist in our preliminary experiment.

As a negative regulator, interleukin (IL)-10 is primarily produced by Th2 cells, activated B cells, monocytes, macrophages, and glial cells (Jesus et al., 2013) and regulates pleiotropic effects in inflammation and immunoregulation (Asadullah et al., 2003; Jankovic et al., 2010), by binding to the IL-10 receptor-α followed by activating IL-10 receptor-β localized on the cell membrane (Scumpia and Moldawer, 2005; Sabat et al., 2010). IL-10 exhibits remarkable neuroprotective and antinociceptive effects in the central nervous system (Bastien and Lacroix, 2014). It has been demonstrated that IL-10 inhibited proinﬂammatory cytokine production, attenuated thermal hyperalgesia induced by chronic sciatic nerve constriction (Wagner et al., 1998), and enhanced morphine analgesia (Johnston et al., 2004). Local injection of IL-10 eliminated mechanical hyperalgesia caused by carrageenan in hindpaw of rats (Poole et al., 1995), and its trigeminal ganglia injection decreased trigeminal neuropathic pain induced by infraorbital nerve constriction in rats (Iwasa et al., 2019). Intrathecal injection of IL-10 produced mechanical antiallodynia and thermal antihyperalgesia through spinal microglial expression of β-endorphin (Wu et al., 2018a). Furthermore, the spinal glial IL-10 and β-endorphin pathway has been revealed to be associated with the antinociceptive effects of electroacupuncture and the agonists of the glucagon-like peptide-1 (GLP-1) receptor and G protein-coupled receptor 40 (GPR40) in rodent models of neuropathic pain induced by spinal nerve ligation (Wu et al., 2017b; Mao et al., 2019; Ali et al., 2020).

α7 nAChRs have been recognized to mediate long-term modification of cell functions, in addition to the typical ligand-gated ion channels that evoke cation-selective currents across the plasma membrane. Sustained stimulation of α7 nAChRs mostly expressed in the central nervous system induces delayed cellular responses leading to neuroprotection and antinociception via intracellular signal pathways probably triggered by Ca^2+^ influx, in addition to acute responses (Kihara et al., 2001). α7 nAChRs are expressed not only in neurons but also in nonneuronal cells such as astrocytes, microglia, oligodendrocyte precursor cells, and brain endothelial cells (Hawkins et al., 2005; Suzuki et al., 2006; Liu et al., 2015). They have recently attracted more attention because their mechanisms of action are involved in neuroinflammation (King et al., 2017), neurodegenerative diseases such as Alzheimer’s disease (Parri et al., 2011), and neuroprotection (Morioka et al., 2015; Xu et al., 2019). Downregulation of spinal α7 nAChR expression is observed in spared nerve injury- and chronic sciatic nerve constriction-induced neuropathic pain (Wang et al., 2018). Furthermore, activation of α7 nAChRs attenuates inflammatory pain, postoperative pain, neuropathic pain, and bone cancer pain (Bagdas et al., 2011; Kiguchi et al., 2012; Gong et al., 2015; Bagdas et al., 2016; Ji et al., 2019; Sun et al., 2019). It was suggested that activation of α7 nAChRs blocked neuropathic pain through promoting expression of the anti-inflammatory cytokine IL-10 and inhibiting expression of proinflammatory cytokines (Arredondo et al., 2006; Zhang et al., 2017). It was also recently revealed that activation of α7 nAChRs produced antinociception in rat models of neuropathic pain and bone cancer pain via spinal microglial expression of IL-10 and β-endorphin (Apryani et al., 2020; Wang et al., 2020).

In this study, we extended our previous research on cynandione-A-mediated antinociception to explore the molecule mechanisms underlying cynandione-A-induced mechanical antiallodynia in neuropathic rats and cultured primary microglial cells. Our results demonstrated that cynandione A produces mechanical antiallodynia in neuropathic pain through spinal microglial IL-10 expression via the cAMP/PKA/p38/CREB signaling and subsequent β-endorphin expression via the IL-10/STAT3 signaling in an α7-nAChR-dependent manner.

## Materials and Methods

### Chemicals and Reagents

Cynandione A was extracted, isolated, and purified by the Institute of Botany, Jiangsu Province and Chinese Academy of Sciences, with ≥98% purity determined by ^1^H-NMR and HPLC. The rabbit β-endorphin antiserum and recombinant rat IL-10 antibody were purchased from Abcam (Cambridge, United Kingdom) and R&D systems (Minneapolis, MN, United States) respectively. The adenylate cyclase inhibitor 2,5-dideoxyadenosine (DDA) and PKA activation inhibitor H-89 were obtained from Santa Cruz Biotechnologies (Santa Cruz, CA, United States), while the p38 activation inhibitor SB203580, CREB activation inhibitor KG501, and STAT3 activation inhibitor NSC74859 were purchased from Selleck Chemicals (Houston, TX, United States), Sigma-Aldrich (St. Louis, MO, United States), and Medchem Express (Boston, United States), respectively. The α7 nAChR antagonist methyllycaconitine citrate was purchased from APEx BIO (Houston, United States). Cynandione A was dissolved in 10% dimethyl sulfoxide (DMSO) and 20% polyethylene glycol (PEG400) in 0.9% normal saline for intrathecal injection and dissolved in 0.1% DMSO for cell culture. All other drugs or reagents were dissolved or diluted in normal saline.

### Animals

Male adult (160–180 g body weight) and 1-day-old (sex unidentified) neonatal Wistar rats were obtained from the Shanghai Experimental Animal Institute for Biological Sciences (Shanghai, China). The adult animals were housed (3–4 per cage) in the Shanghai Jiao Tong University Experimental Animal Center (Shanghai, China) in room temperature (22 ± 2°C) under light conditions of a 12/12 h reversed light-dark cycle (7:00 a.m.-7:00 p.m.). They received food and water *ad libitum*, accustomed to the laboratory environment for 3–4 days before surgery. The research protocols were approved by the Animal Care and Welfare Committee of Shanghai Jiao Tong University and carried out in accordance with the Animal Care Guidelines of the US National Institutes of Health.

### Primary Cultures of Microglia

Primary microglial cells were isolated from the spinal cords of 1-day-old neonatal rats as previously reported (Mao et al., 2019). The isolated spinal cords were minced and incubated in 0.05% trypsin in the incubator for 7 min. The digestion was terminated using the DMEM supplemented with 10% (v/v) fetal bovine serum (FBS), penicillin (100 U/mL), and streptomycin (100 mg/ml). The glia cells were then plated into 75-cm^2^ tissue culture flasks (1 × 10^7^ cells/flask) that were precoated with poly-L-lysine (100 mg/ml) and cultured at 37°C in a 5% carbon dioxide incubator. After 8 days of culture, microglial cells were prepared as floating cell suspensions by shaking the flasks at 260 rpm for 2 h. Unattached microglial cells were removed by washing with serum-free DMEM and centrifuged with 300 g for 15 min, and transferred into new 12- or 24-well plates for further study.

### RNA Isolation and Quantitative Reverse Transcriptase-Polymerase Chain Reaction (qRT-PCR)

The spinal lumbar enlargements (L3–L5) were isolated from neuropathic rats and homogenized (4,000 rpm) in the Trizol reagent (Invitrogen, Carlsbad, United States) for 15 s with a homogenizer (Fluko Equipment Co., Shanghai, China). The total RNAs from spinal homogenates and primary microglia were extracted using the Trizol buffer and reversely transcribed into cDNA using the ReverTra Ace qRT-PCR RT-kit (Toyobo Co., Osaka, Japan) according to the manufacturers’ protocols. The qRT-PCR amplification was conducted in a Mastercycler ep realplex (Eppendorf, Germany) using the Realmaster Mix (SYBR Green I; Toyobo Co., Osaka, Japan). The primers used were as follows: 5′-GGC​TCA​GCA​CTG​CTA​TGT​TGC​C-3′ and 5′-AGC​ATG​TGG​GTC​TGG​CTG​ACT​G-3’ (IL-10; NM_012854.2) (Wu et al., 2017b), 5′-CCT​ATC​GGG​TGG​AGC​ACT​TC-3′ and 5′-TGG​CTC​TTC​TCG​GAG​GTC​AT-3’ (*POMC* exon 2–3) (Busch-Dienstfertig et al., 2012; Sitte et al., 2007), 5′-ACT​GCC​TGT​CCT​TGT​GTT​CC-3′ and 5′-CCA​AAG​CAA​CCT​CAT​TCT​CC-3′ for the dynorphin A precursor prodynorphin (PDYN) (Leitl et al., 2014), 5′-CCA​AGG​TCA​TCC​ATG​ACG​AC-3′ and 5′-TCC​ACA​GTC​TTC​TGA​GTG​GC-3’ (*GAPDH*) (Gong et al., 2014). qRT-PCR was first identified to be specific using the melting curves and the relative expression of each mRNA level was calculated with the 2^-∆∆CT^ method after normalizing Ct (cycle threshold) values with GAPDH Ct.

### Western Blot

Protein supernatants, mechanically homogenized from the spinal lumbar enlargements (L3–L5) and cultured microglia, were lysed in the RIPA lysis buffer which contained 1% protease inhibitor PMSF and 1% phosphorylase inhibitors cocktail A/B (Biotool, Houston, United States). The protein solution was denatured at 100°C after adding 5 x of the SDS protein loading buffer and separated in 10% of the sodium dodecyl sulfate—polyacrylamide gel electrophoresis (SDS-PAGE) with the PAGE gel electrophoresis and then transferred to a polyvinylidene fluoride membrane using the electrophoretic method. The membrane was then blocked in 5% skim milk powder dissolved in 1 x TBS containing 0.1% Tween 20 (TBS-T). After blocking for 1 h, the membrane was incubated with the primary antibody against p-STAT3 (phosphor-Stat3, Ser727) (1:1,000; Cell Signaling Technology, Danvers, MA, United States), p-PKA (1:2000; Abcam, Cambridge, United Kingdom), p-p38 (1:1,000; Cell Signaling Technology), p-CREB (1:1,000; Cell Signaling Technology), and GAPDH (1:5,000; Protein Tech Group, Chicago, United States) overnight at 4°C with gentle shaking. After collecting the primary antibody and washing the membrane four times with TBS-T (10 min once) for the second day, the membrane was incubated with the corresponding second antibody diluted in 5% skim milk powder dissolved in 1x TBS-T (1:10,000), including the goat anti-rabbit IgG (IRDye 800-conjugated, Cell Signaling Technology) and goat anti-rat IgG (IRDye 700-conjugated, Cell Signaling Technology) for 1 h at 37°C with gentle shaking. The second antibody was collected and the membrane was washed with 5% skim milk powder dissolved in 1x TBS-T for four times (10 min once). The Odyssey Infrared Imaging system (Li-Cor Biosciences, Lincoln, NE, United States) was used to detect the protein bands, which was analyzed and quantified by using the Image J program (National Institutes of Health, Bethesda, MD, United States). The relative expression of each target protein was obtained after normalization to the GAPDH level as previously reported. The experiments were repeated at least three times.

### Immunofluorescence Staining

Double immunofluorescence labeling of IL-10 and β-endorphin with cellular biomarkers of microglia, astrocytes, and neurons was performed in the spinal cord and visualized under a TCS SP8 confocal microscope (Leica Microsystems, Wetzlar, Germany) as described previously (Huang et al., 2016). Pentobarbital-anesthetized rats (40 mg/kg) were subjected to the intracardial perfusion with 100 ml of normal saline to flush blood of the whole body, followed by 60 ml of 4% paraformaldehyde (w/v) to fix cells in the spinal cord. The rats were sacrificed and the spinal lumbar enlargements (L3–L5) were isolated and fixed in 4% buffered paraformaldehyde for 18 h and dehydrated in gradient sucrose solutions (10–30%) at 4°C. Tissues were entrapped and frozen in the OCT-freeze tissue medium (Leica Microsystems) and cut into 30-μm sections. The frozen sections were blocked by 10% goat serum (v/v) and 0.5% X-100 (v/v) in phosphate-buffered saline (PBS) at room temperature for 1 h after washing in PBS and baking in a 37°C-dryer machine for 30 min to prevent tissue detachment. The sections were then incubated with the IL-10 antibody (1:100; goat polyclonal; R&D Systems) or β-endorphin antiserum (1:100; rabbit polyclonal; Phoenix Pharmaceuticals) with the primary antibodies against cellular biomarkers at 4°C for 24 h. The cellular biomarker antibodies included Iba-1 for microglia (1:100; mouse monoclonal; Millipore, United States), GFAP for astrocytes (1:100; mouse polyclonal; Millipore), and NeuN for neurons (1:60; mouse polyclonal; Millipore). The IL-10 or β-endorphin staining was visualized with the Alexa Fluor-555-conjugated donkey anti-goat secondary antibody (1:200; Invitrogen, California, United States) or Alexa Fluor-555-conjugated goat anti-rabbit secondary antibody (1:200; Invitrogen), while the Alexa Fluor-488-conjugated goat anti-mouse secondary antibody (1:200; Invitrogen) was used to detect the cell biomarkers.

For quantification of the intensity of IL-10-, β-endorphin-, Iba-1-, GFAP-, NeuN-, and α7 nAChR-positive cells, photomicrographs of the medial three-fourths of the dorsal horn (laminas I-III) and cultured microglial cells (see below) were taken under a confocal microscope with ×10 or ×30 magnification. An investigator blinded to the experimental groups measured the positively stained surface area using the computer-assisted image analysis program, Image J software (National Institutes of Health, United States). The background fluorescence was excluded and only immunofluorescence intensity measurements from positive-staining areas were included by low- and high-threshold setup. For the colocalization analysis, the colocalization finder of the Image J software was used to generate merged images in which colocalized pixels appeared as white.

For the staining and quantification, the cultured primary microglial cells were seeded on the poly L-lysine-coated round coverslips which were placed in the 24-well plates (1 × 10^4^/well) and cultured overnight. The cells were then washed with PBS 2 h after cynandione A treatment and fixed in 4% paraformaldehyde for at least 1 h, then the coverslips moved in the 12-well plates were incubated in 10% goat serum (v/v) and 0.5% X-100 (v/v) in PBS for blocking in room temperature for 1 h. The rest of procedures followed the above protocol of the frozen spinal sections except that cultured microglial cells were also stained with the nucleic dye reagent 2-(4-amidinophenyl)-6-indolecarbamidine dihydrochloride (DAPI, 0.1 μg/ml; Beyotime Biotechnology, Shanghai, China) for 3 min and were taken under a confocal microscope with ×30 or ×60 magnification.

### IL-10/β-Endorphin Measurements

The contralateral and ipsilateral spinal lumbar enlargements (L3–L5) were isolated from neuropathic rats 1 h after intrathecal drug injection and homogenized at 4,000 rpm for 15 s with a homogenizer (Fluko Equipment, Germany) in 10 mM Tris-HCl (5 ml/1 g of tissue) and centrifuged at 4,000 rpm at 4°C for 15 min. In addition, cultured primary microglial cells were placed in 24-well plates (1 × 10^5^ cells/well) and washed once with 1 ml/well of warm PBS, twice with 1 ml/well of warm DMEM containing 2 mg/ml BSA and 15 mmol/L *N*-(2-hydroxyethyl) piperazine-N-2-ethanesulfonic acid and then incubated with 100 μM cynandione A for 2 h according to the previous study (Huang et al., 2017). The cell culture supernatant was collected and further centrifuged at 5,000 rpm for 10 min at 4°C and then aspirated to a new tube. The total protein concentrations in the spinal cord homogenates were measured using the standard bicinchoninic acid protein assay (Beyotime Biotechnology, Shanghai, China). The levels of IL-10 (eBioscience, California, United States) and β-endorphin (Phoenix Pharmaceuticals, United States) were measured using the commercial fluorescent immunoassay kits. A microplate reader (Multiskan MK3; Thermo Labsystems, Vantaa, Finland) and a fluorescence microplate reader (Thermo Labsystems, Grand Rapids, Wood Ohio, United States) were used to measure the relative fluorescence values and the concentrations of IL-10/β-endorphin were calculated by a calibration curve performed at the same time. The assays were validated with the linear range of 1–500 and 1–100 pg/ml for the IL-10 and β-endorphin, respectively.

### Rat Models of Neuropathic Pain and Intrathecal Catheterization

Intrathecal catheterization was performed in rats according to the previously described protocol (Huang et al., 2012). Briefly, a 20-cm catheter (PE-10: 0.28 mm inner diameter and 0.61 mm outer diameter, AniLab Software and Instruments Co., Ningbo, China) was inserted into the lumbar level of the spinal cord under inhaled isoflurane anesthesia. The other end of the PE-10 catheter was inserted subcutaneously to the neck and fixed. The spinal nerve ligation procedure was performed at the same time just after intrathecal catheterization. The left L5 and L6 spinal nerves were carefully isolated and tightly ligated with 6–0 silk sutures. The lumbar fascia and skin were sewed by a 4–0 absorbable polyglactin suture after nerve ligation. The rat returned to its single home cage after surgeries for recovery. Only rats with no major motor impairments and significant unilateral allodynia to mechanical stimulation (hindpaw withdrawal thresholds in the operated side <8 g) and with both hindpaws of immediate limp and feeble after intrathecal injection of 10 μl of 4% lidocaine followed by 15 μl saline flush were chosen for subsequent experiments. Neuropathic rats underwent different drug tests during 1–2 weeks after spinal nerve ligation.

### Mechanical Allodynia Assessment

The hindpaw withdrawal threshold to mechanical stimuli was measured using a 2290CE electrical von Frey hair (IITC Life Science Inc., CA, United States) according to the previous study (Wang et al., 2017). Neuropathic rats were acclimatized to the Plexiglas box on a metal grid for at least 30 min. An examiner blinded to the treatment groups performed the behavior testing using a 2290 CE electrical von Frey hair (IITC Life Science, Woodland Hill, CA, United States). The withdrawal thresholds were evoked in both contralateral and ipsilateral hindpaws using the von Frey hair while the rat stood on a metal grid. The increments of force were applied to stimulate the footpad until the rat suddenly withdrew its hindpaw. The lowest force evoking a withdrawal response was considered the threshold, which was averaged from triplicate measurements at a 1-min interval.

### Data Statistical Analysis

Data were exhibited as means ± SEM. Two-tailed and unpaired Student t-test and one-way or repeated measures two-way ANOVA were applied to generate statistical significance values. The post-hoc Student-Newman-Keuls test was used when the effect of the drug (dose) (for one-way ANOVA, the factor was drug [dose]; for two-way ANOVA, the factors were drug [dose], time, and their interaction) was statistically significant. The statistical analysis was performed using GraphPad Prism (Version 7.0, GraphPad Software, San Diego, CA, United States). Probability values were considered statistically significant at 5% level.

## Results

### Cynandione A Produced Mechanical Antiallodynia in Neuropathic Pain and Specifically Stimulated Spinal Microglial Expression of IL-10 and β-Endorphin

**FIGURE 1:**
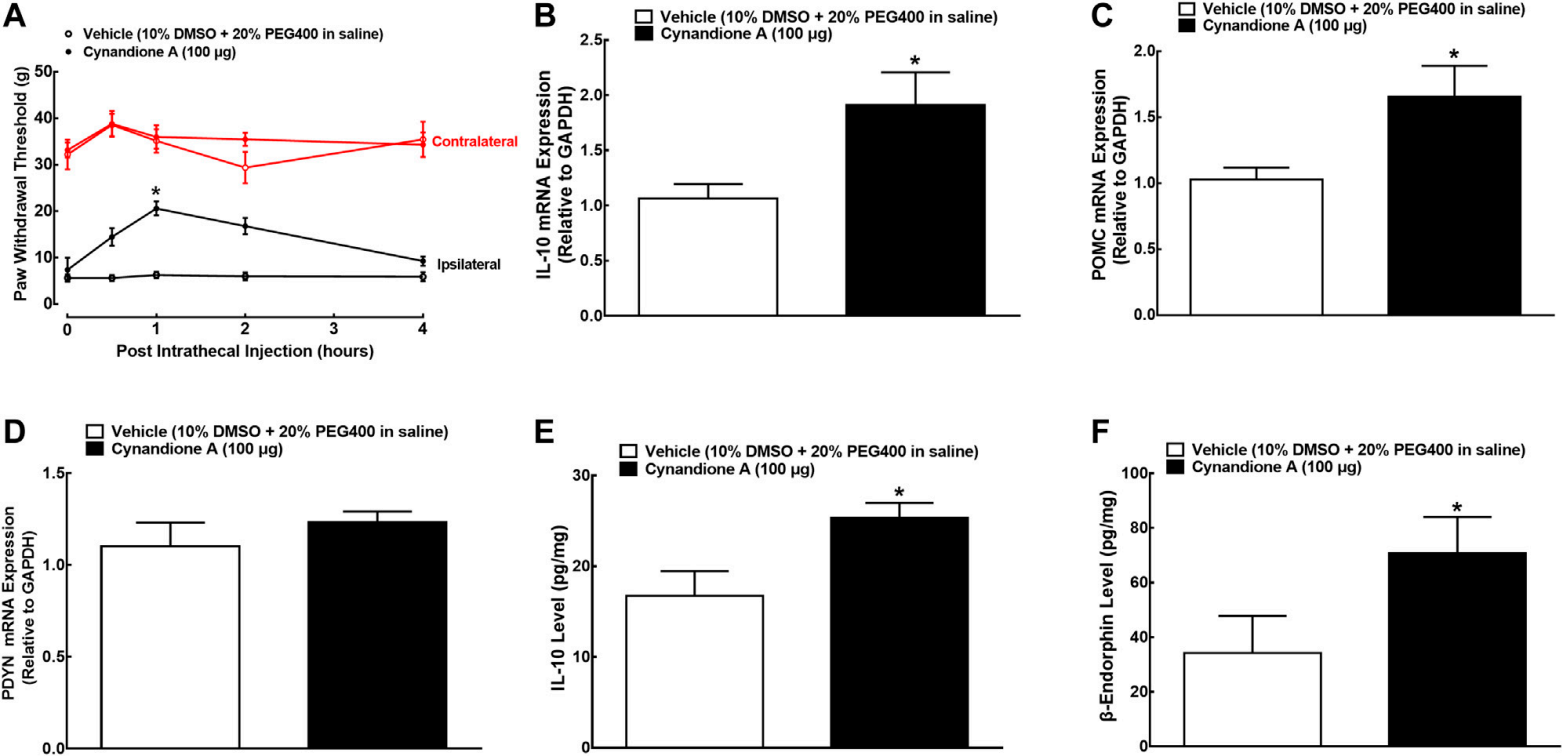
Effects of cynandione A, given intrathecally, on mechanical allodynia **(A)**, spinal mRNA expression of IL-10 **(B)**, the β-endorphin precursor proopiomelanocortin (POMC **(C)**) and dynorphin precursor prodynorphin (PDYN **(D)**), and spinal protein expression of IL-10 **(E)** and β-endorphin **(F)** in neuropathic rats induced by L5/L6 spinal nerve ligation. Spinal lumbar enlargements (L3-L5) were obtained from the sacrificed rats 1 h after injection of cynandione A or the vehicle. The gene and protein expression was determined by using qRT-PCR and enzyme-linked immunosorbent fluorescent assays, respectively. Data are shown as means ± SEM (*n* = 6 per group). ^*^ *p* < 0.05 compared with the vehicle group, analyzed by two-tailed and unpaired Student t-test or repeated measures two-way ANOVA followed by the post-hoc Student-Newman-Keuls test.

We have previously demonstrated that intrathecal injection of cynandione A dose-dependently attenuated mechanical allodynia and thermal hyperalgesia in neuropathic pain, with E_max_ values of 57 and 59% maximum possible effect and ED_50_ values of 14.9 and 6.5 μg, respectively (Huang et al., 2017). In this study, we determined its spinal mechanical antiallodynic effect at 100 μg, an approximately ED_90_ of cynandione A (Huang et al., 2017). Two groups of spinal nerve ligated neuropathic rats (*n* = 6 per group) received single intrathecal injection of 10 μl of the vehicle (10% DMSO and 20% PEG400 in saline) or 100 μg of cynandione A. The withdrawal thresholds in both contralateral and ipsilateral hindpaws of the vehicle-treated control rats were unchanged during the 4-h observation. Intrathecal injection of cynandione A did not significantly alter withdrawal thresholds in the contralateral hindpaws, but time dependently inhibited mechanical allodynia in the ipsilateral hindpaws with the peak effect at 1 h by 48% MPE and duration of approximately 4 h after injection (*P* < 0.05, by repeated measures two-way ANOVA followed by the post-hoc Student-Newman-Keuls test; Figure 1A).

Additional two groups of neuropathic rats (*n* = 6 per group) that received the same intrathecal treatments as above were sacrificed 1 h after injection (peak time of the antiallodynic effect). The spinal cords were collected and homogenized to detect the gene and protein expression of IL-10, β-endorphin and dynorphin A by using qRT-PCR and immunoassay kits, respectively. As shown in Figures 1B–D, intrathecal injection of cynandione A (100 μg) specifically stimulated spinal mRNA expression of IL-10 and POMC (P< 0.05, by unpaired and two-tailed Student t-test) but not PDYN. Moreover, intrathecal cynandione A also significantly stimulated spinal protein expression of IL-10 and β-endorphin (*P* < 0.05, by unpaired and two-tailed Student t-test; Figures 1E,F).

**FIGURE 2:**
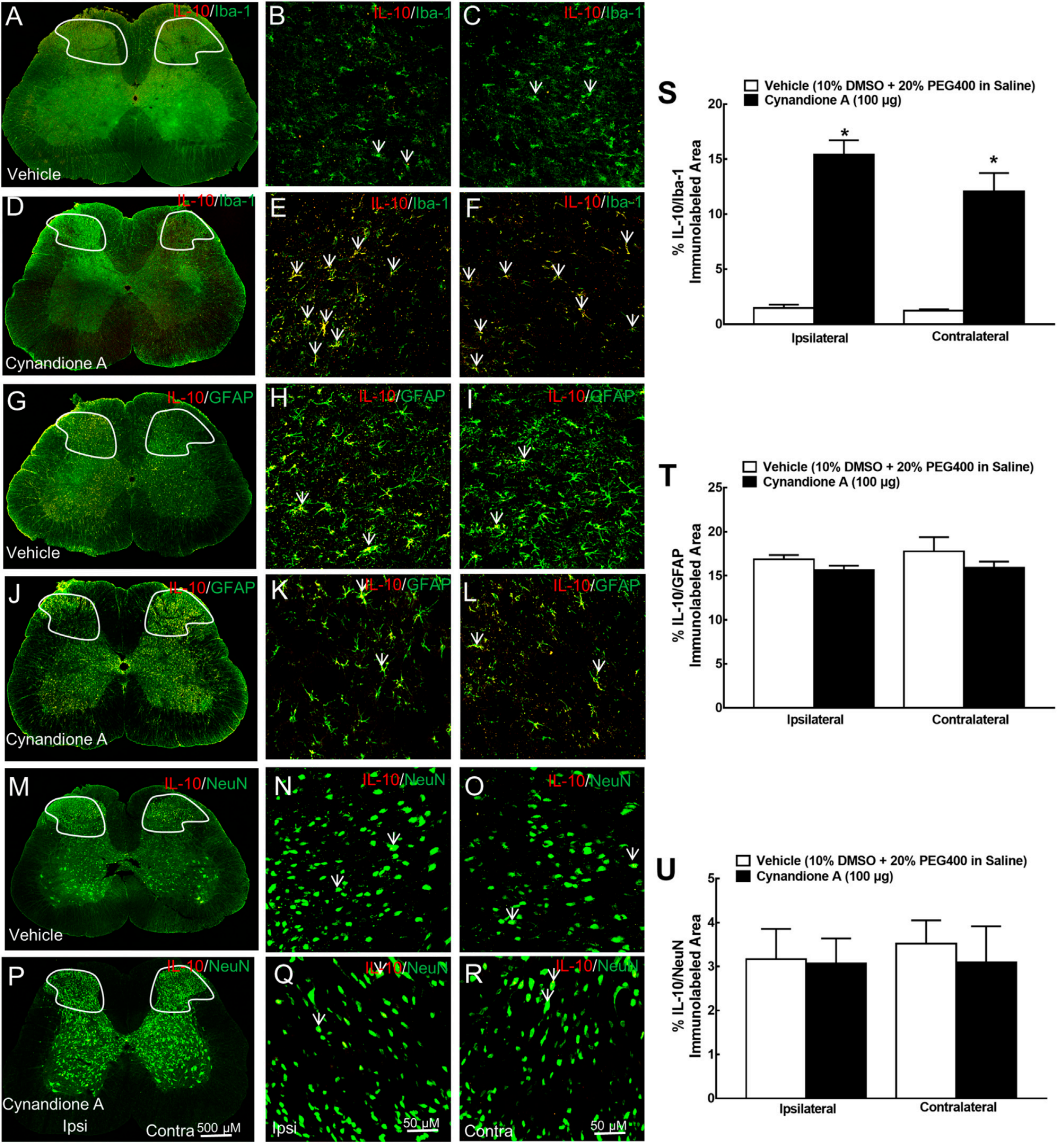
Specific stimulatory effects of cynandione A, given intrathecally, on spinal microglial expression of IL-10 in neuropathic rats induced by tight ligation of L5/L6 spinal nerves. Neuropathic rats that received intrathecal injection of cynandione A or the vehicle were sacrificed 1 h after injection and the spinal lumbar enlargements (L3–L5) were obtained. The specific expression of IL-10 was double fluorescence immunolabeled with the microglial cellular biomarker Iba-1 **(A–F)**, astrocytic cellular biomarker GFAP **(G-L),** and neuronal cellular biomarker NeuN **(M–R)** in the contralateral and ipsilateral spinal cord and dorsal horn I-Ⅲ laminate. Arrows indicate yellow double labeling of IL-10 with the corresponding cellular biomarkers. The immunolabeled surface areas of IL-10/Iba-1 **(S)**, IL-10/GFAP **(T)**, and IL-10/NeuN **(U)** from the indicated spinal dorsal horn laminae I-Ⅲ were quantified using the Image J program. Data are presented as means ± SEM (*n* = 5 per group). ^*^ *P* < 0.05 compared with the vehicle group, analyzed by one-way ANOVA followed by the post-hoc Student-Newman-Keuls test.

To further test the specific expression of spinal microglial IL-10 and β-endorphin, their double immunofluorescence labeling was performed with cellular biomarkers of microglia (Iba-1), astrocytes (GFAP), or neurons (NeuN). Two groups of neuropathic rats (*n* = 5 per group) received intrathecal injection of 10 μL of the vehicle or 100 μg of cynandione A. The rats were sacrificed 1 h after injection and the spinal cords were collected for immunostaining. There was no significant difference of the double IL-10/Iba-1 immunostaining between contralateral and ipsilateral spinal dorsal horns observed under ×10 or ×30 magnifications. Cynandione A treatment significantly enhanced the double IL-10/Iba-1 immunostaining in both contralateral and ipsilateral spinal dorsal horns compared to the vehicle control (Figures 2A–F). In contrast, intrathecal cynandione A injection did not enhance double immunostaining of IL-10/GFAP (2G-2L) or IL-10/NeuN (2M-2R). As revealed by a confocal microscope with ×30 magnification, cynandione A in the contralateral and ipsilateral dorsal horn I-III laminate significantly increased double immunofluorescence intensity of IL-10/Iba-1 by 8.8-fold and 9.5-fold, respectively (*p* < 0.05, by one-way ANOVA followed by the post-hoc Student-Newman-Keuls test; Figure 2S), but not double immunofluorescence intensity of IL-10/GFAP (Figure 2T) or IL-10/NeuN (Figure 2U).

**FIGURE 3:**
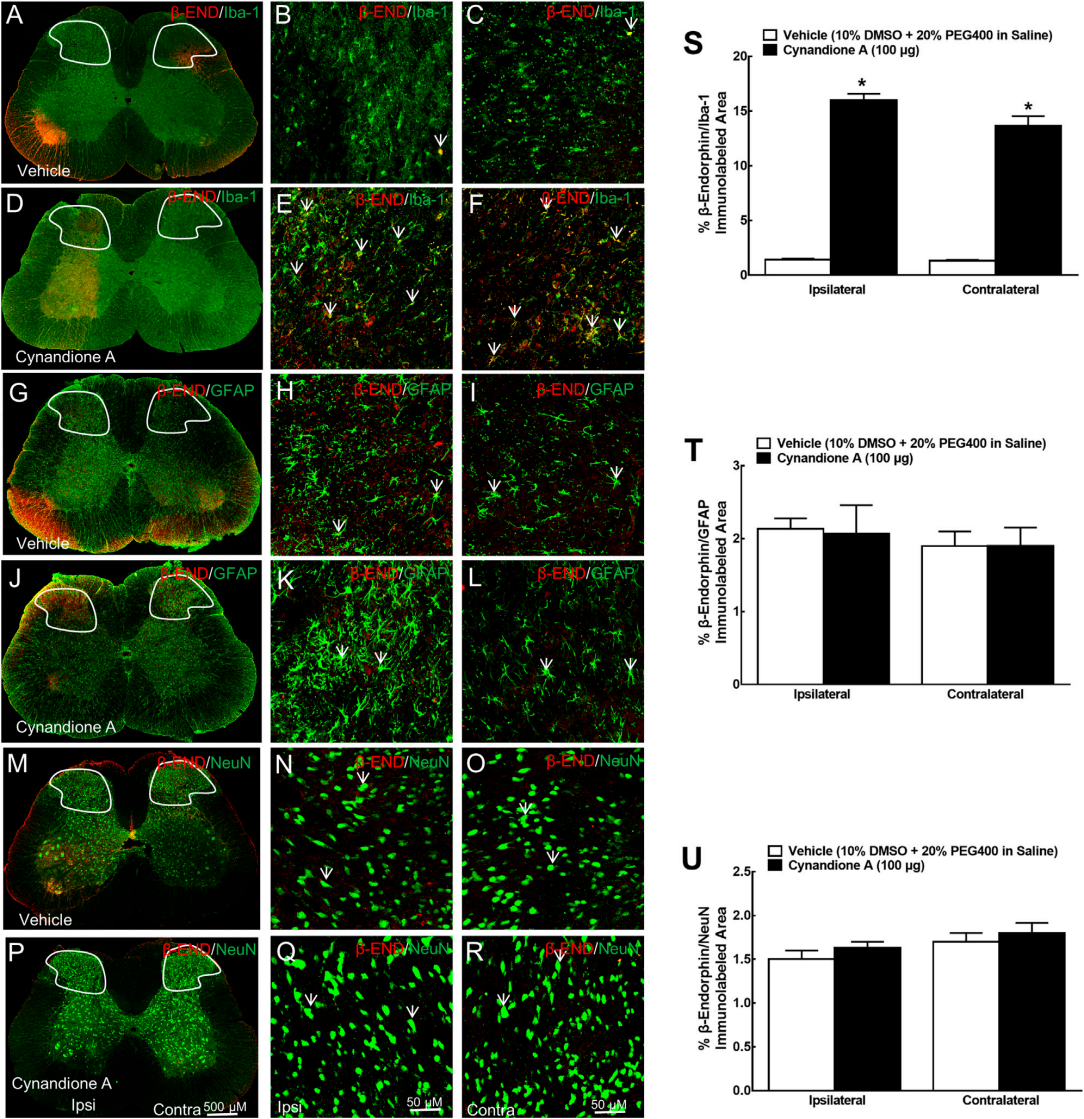
Specific stimulatory effects of cynandione A, given intrathecally, on spinal microglial expression of β-endorphin in neuropathic rats induced by tight ligation of L5/L6 spinal nerves. Neuropathic rats that received intrathecal injection of cynandione A or the vehicle were sacrificed 1 h after injection and the spinal lumbar enlargements (L3–L5) were obtained. Expression of β-endorphin was double fluorescence immunolabeled with the microglial cellular biomarker Iba-1 **(A–F)**, astrocytic cellular biomarker GFAP **(G-L),** and neuronal cellular biomarker NeuN **(M–R)** in the contralateral and ipsilateral spinal cord and dorsal horn I-Ⅲ laminate. Arrows indicate yellow double labeling of β-endorphin with the corresponding cellular biomarkers. The immunolabeled surface areas of β-endorphin/Iba-1 **(S)**, β-endorphin/GFAP **(T),** and β-endorphin/NeuN **(U)** from the indicated spinal dorsal horn laminae I-Ⅲ were quantified using the Image J program. Data are presented as means ± SEM (*n* = 5 per group). ^*^ *P* < 0.05 compared with the vehicle group, analyzed by one-way ANOVA followed by the post-hoc Student-Newman-Keuls test.

Furthermore, the specific stimulatory effect of intrathecal cynandione A on β-endorphin expression was also demonstrated in microglia but not astrocytes or neurons in the spinal dorsal horn (Figure 3A-3R). Quantitative measurement indicated that cynandione A increased the double immunofluorescence intensity of β-endorphin/Iba-1 in the contralateral and ipsilateral dorsal horn I-III laminate by 9.3-fold and 10.2-fold, respectively (*p* < 0.05. by one-way ANOVA followed by the post-hoc Student-Newman-Keuls test; Figure 3S), but not the double immunofluorescence intensity of β-endorphin/GFAP (Figure 3T) or β-endorphin/NeuN (Figure 3U).

### Cynandione A Stimulated IL-10 and β-Endorphin Expression in Primary Spinal Microglia

**FIGURE 4:**
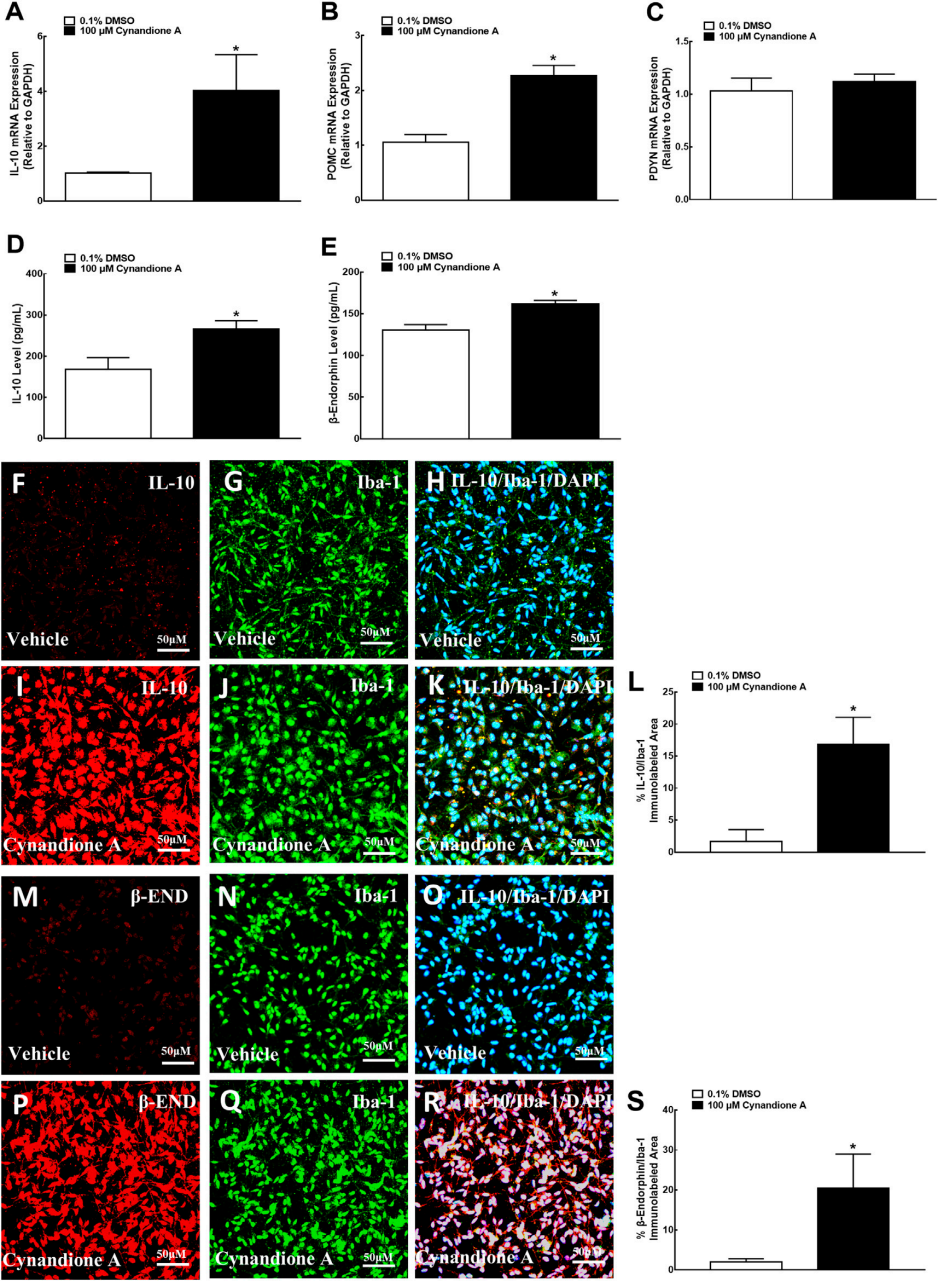
Effects of cynandione A on gene **(A–C)** expression, protein secretion **(D, E),** and fluorescent immunostaining **(F–S)** of IL-10, β-endorphin, and dynorphin A in primary cultures of spinal microglia collected from 1-day-old neonatal rats. Primary microglial cells were incubated with cynandione A for 2 h. The qRT-PCR and enzyme-linked immunosorbent fluorescent kits were used to detect gene expression and protein secretion in the cells and culture medium, respectively. Single and triple immunofluorescence staining was used to detect I-10 (red), Iba-1 (green), and IL-10 (red)/Iba-1 (green)/DAPI (blue) or β-endorphin (red)/Iba-1 (green)/DAPI (blue). The immunolabeled surface areas of IL-10/Iba-1 (R) and β-endorphin/Iba-1 (S) were quantified by using the Image J program. The data are presented as means ± SEM (*n* = 3 per group with two independent repeats). ^*^ *p* < 0.05 compared with the vehicle group, by two-tailed and unpaired Student t-test.

We have previously demonstrated that treatment with cynandione A (3, 10, 30, 100, and 300 μM) concentration dependently stimulated POMC and β-endorphin expression in cultured microglia (but not astrocytes or neurons), with EC_50_ values of 38.8 and 20.0 μM, respectively (Huang et al., 2017). In this study we first test its stimulatory effects on IL-10 and β-endorphin at 100 μM, an approximately EC_80_ of cynandione A, in cultured microglial cells originated from neonatal rats. The cultured cells were collected 2 h later and digested to detect the gene expression of IL-10, β-endorphin and dynorphin A by using qRT-PCR, while the cell culture medium was collected to detect the protein expression of IL-10 and β-endorphin by using commercial fluorescent immunoassays. As shown, treatment with cynandione A in microglia significantly upregulated the mRNA expression of IL-10 and POMC (*P* < 0.05, by two-tailed and unpaired Student t-test; Figures 4A,B), but not PDYN (Figure 4C). In addition, cynandione A treatment upregulated IL-10 and β-endorphin levels in the cell culture medium (*P* < 0.05, by two-tailed and unpaired Student t-test; Figures 4D,E).

The stimulatory effect of cynandione A on IL-10 and β-endorphin was further assessed in cultured microglia by using single and triple immunofluorescence labeling of IL-10 or β-endorphin with Iba-1 and the nuclear staining reagent DAPI. Compared with the vehicle control, treatment with cynandione A (100 μM) significantly enhanced the IL-10 expression reflected in single or triple immunostaining under ×30 magnifications (Figures 4F–K). Quantitatively, cynandione A treatment significantly increased triple immunofluorescence intensity of IL-10/Iba-1/DAPI by 9.0-fold (p< 0.05, by one-way ANOVA followed by the post-hoc Student-Newman-Keuls test; Figure 4L).

In addition, cynandione A (100 μM) treatment also significantly stimulated the β-endorphin expression in both single and double immunostaining (Figures 4M–R). Quantitative measurement revealed that cynandione A significantly increased double immunofluorescence intensity of β-endorphin/Iba-1 by 9.0-fold (*p* < 0.05, by one-way ANOVA followed by the post-hoc Student-Newman-Keuls test; Figure 4S).

### Cynandione A Produced Mechanical Antiallodynia in Neuropathic Pain Through Spinal Microglial IL-10 Expression and Subsequent β-Endorphin Expression

**FIGURE 5:**
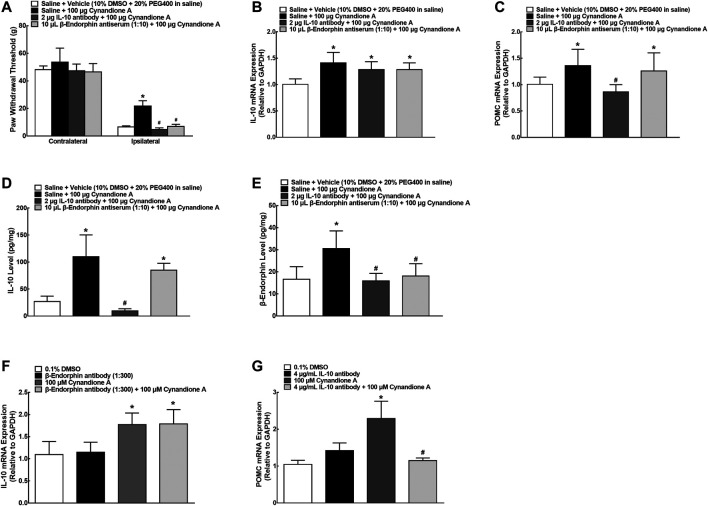
Blockade effects of the IL-10 antibody and β-endorphin antiserum on cynandione-A-induced spinal mechanical antiallodynia **(A)**, spinal gene expression of IL-10 **(B)** and the β-endorphin precursor proopiomelanocortin (POMC **(C)**), and spinal protein expression of IL-10 **(D)** and β-endorphin **(E)** in neuropathic rats induced by L5/L6 spinal nerve ligation. Neuropathic rats received intrathecal injection of saline, the IL-10 antibody or β-endorphin antiserum followed by intrathecal injection of the vehicle or cynandione A 30 min later. The spinal cords were obtained immediately after the completion of behavioral test (1 h after the last injection) for detection of IL-10 and β-endorphin by using qRT-PCR and enzyme-linked immunosorbent fluorescent assays, respectively. Blockade effects of the IL-10 antibody and β-endorphin antiserum on cynandione-A-stimulated gene expression of POMC **(F)** and IL-10 **(G)** in cultured microglial cells originated from 1-day-old neonatal rats. Data are shown as means ± SEM (*n* = 6 per group in neuropathic rats or *n* = 3 per group with two independent repeats in cultured cells). *, ^#^ *p* < 0.05 compared with the control and cynandione A treatment groups, respectively, analyzed by one-way ANOVA followed by the post-hoc Student-Newman-Keuls test.

We further explored the causal relationship between spinal microglial expression of IL-10/β-endorphin and mechanical antiallodynia in neuropathic pain. Four groups of neuropathic rats (*n* = 6 per group) received intrathecal injection of saline (10 μl), the IL-10 neutralizing antibody (2 μg) or β-endorphin antiserum (1:10) followed by intrathecal injection of the vehicle (10 μl) or cynandione A (100 μg) 30 min later. The withdrawal thresholds in the contralateral and ipsilateral hindpaws were measured 1 h after injection. Intrathecal injection of cynandione A inhibited mechanical allodynia in the ipsilateral hindpaws, which was nearly completely blocked by the pretreatment with intrathecal injection of the IL-10 antibody or β-endorphin antiserum (*P* < 0.05, by one-way ANOVA followed by the post-hoc Student-Newman-Keuls test; Figure 5A). Pretreatment with intrathecal injection of the IL-10 antibody or β-endorphin antiserum did not significantly affect the baseline mechanical thresholds in the ipsilateral hindpaws as previously reported (Wu et al., 2017b; Huang et al., 2017).

The above four groups of neuropathic rats were sacrificed immediately after the completion of the behavior testing and the spinal cords were collected and homogenized to detect the gene and protein expression of IL-10 and β-endorphin by using qRT-PCR and fluorescent immunoassays, respectively. As shown in Figure 5B, intrathecal cynandione A specifically stimulated spinal mRNA expression of IL-10, which was not significantly reduced by intrathecal injection of the IL-10 antibody or β-endorphin antiserum. On the other hand, cynandione A also stimulated spinal mRNA expression of POMC, which was completely attenuated by the pretreatment with intrathecal injection of the IL-10 antibody (*P* < 0.05, by one-way ANOVA followed by the post-hoc Student-Newman-Keuls test) but not the β-endorphin antiserum (Figure 5C). In addition, as shown in Figures 5D,E, intrathecal injection of cynandione A stimulated IL-10 and β-endorphin expression; pretreatment with the IL-10 antibody neutralized IL-10 secreted and inhibited expression of β-endorphin (*P* < 0.05, by one-way ANOVA followed by the post-hoc Student-Newman-Keuls test). However, pretreatment with intrathecal injection of the β-endorphin antiserum neutralized β-endorphin (but not IL-10) secreted (*P* < 0.05, by one-way ANOVA followed by the post-hoc Student-Newman-Keuls test).

Furthermore, microglial cells from neonatal rats were treated with the IL-10 antibody (4 μg/ml) or β-endorphin antiserum (1:300) for 0.5 h before cynandione A (100 μM) treatment over 2 h. As shown in Figures 5F,G, treatment with cynandione A stimulated the mRNA expression of IL-10 and POMC in cultured microglial cells. Pretreatment with the IL-10 antibody did not significantly alter cynandione-A-stimulated IL-10 mRNA expression but completely reduced its stimulation on POMC expression (*P* < 0.05, by one-way ANOVA followed by the post-hoc Student-Newman-Keuls test). On the other hand, pretreatment with the β-endorphin antiserum did not significantly alter mRNA expression of IL-10 or POMC.

### Cynandione A Produced Mechanical Antiallodynia in Neuropathic Pain and Stimulated IL-10/β-Endorphin Expression in Spinal Microglia in an α7-nAChR-Dependent Manner

**FIGURE 6:**
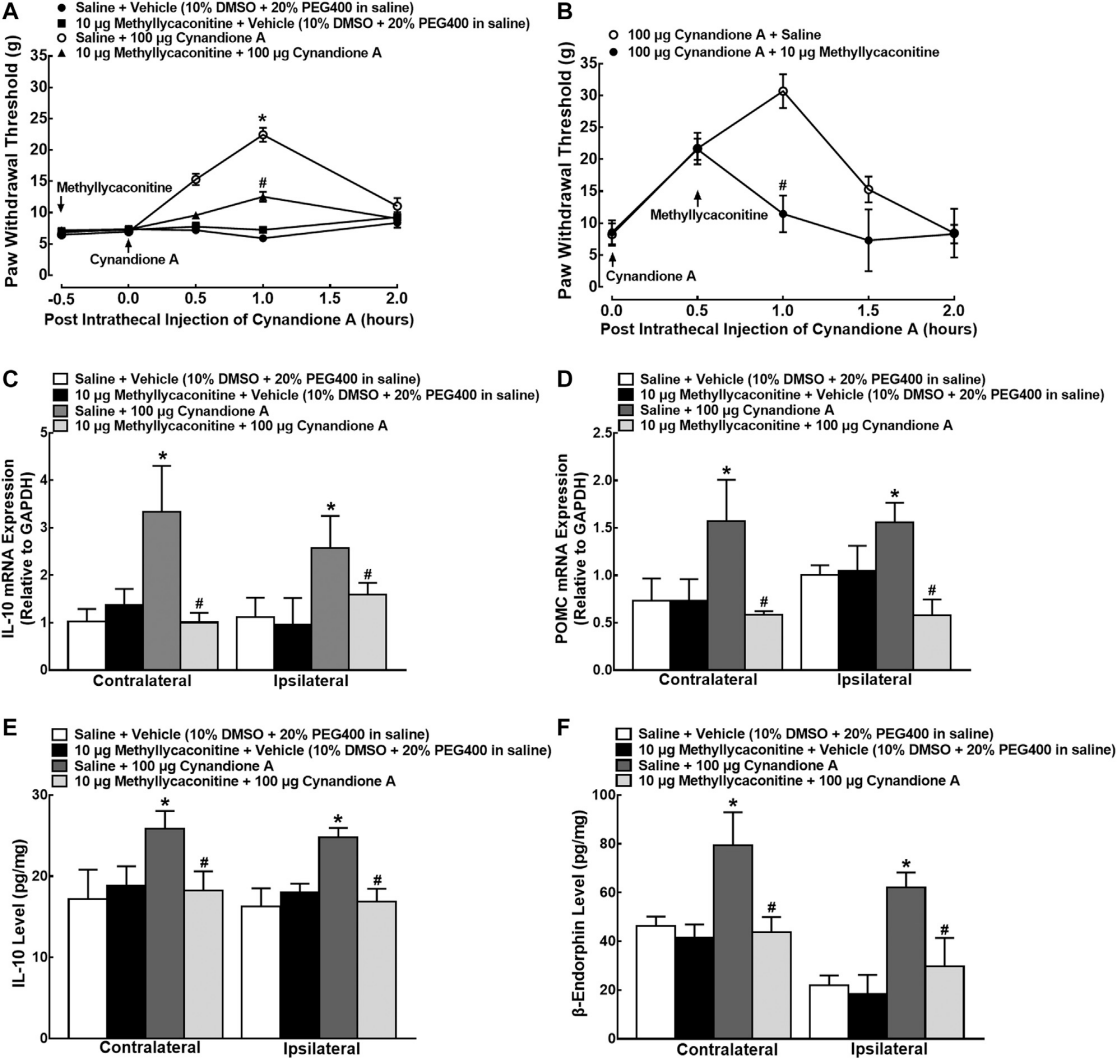
Blockade effects of the α7 nicotinic acetylcholine receptor (α7 nAChR) antagonist methyllycaconitine, given before or after cynandione A injection, on cynandione-A-induced spinal mechanical antiallodynia in the ipsilateral hindpaws **(A,B)**, spinal gene expression of IL-10 **(C)** and the β-endorphin precursor proopiomelanocortin (POMC **(D)**), and protein expression of IL-10 **(E)** and β-endorphin **(F)** in neuropathic rats induced by spinal nerve ligation. Neuropathic rats received intrathecal injection of the vehicle or methyllycaconitine followed by intrathecal saline or cynandione A 30 min later. The spinal cords were obtained 1 h after the last intrathecal injection for detection of IL-10 and β-endorphin by using qRT-PCR and enzyme-linked immunosorbent fluorescent assays, respectively. For the postinjection study, neuropathic rats received intrathecal injection of cynandione A followed by methyllycaconitine 30 min later. Data are shown as means ± SEM (*n* = 6 per group in neuropathic rats). *, ^#^ *p* < 0.05 compared with the control and cynandione A treatment groups, respectively, analyzed by one-way or measures-repeated two-way ANOVA followed by the post-hoc Student-Newman-Keuls test.

In order to illustrate whether cynandione-A-induced mechanical antiallodynia was through activation of α7 nAChRs, four groups of neuropathic rats (*n* = 6 per group) received intrathecal saline (10 μl) or the specific α7 nAChR antagonist methyllycaconitine (10 μg) (Escubedo et al., 2005), followed by intrathecal injection of the vehicle (10 μl) or cynandione A (100 μg) 30 min later. Intrathecal injection of cynandione A in the ipsilateral hindpaws produced time-dependent mechanical antiallodynia, which was nearly completely inhibited by intrathecal preinjection of methyllycaconitine (*p* < 0.05, by repeated measures two-way ANOVA followed by the post-hoc Student-Newman-Keuls test), although it did not significantly alter baseline mechanical thresholds in both contralateral and ipsilateral hindpaws (Figure 6A). In addition, two groups of neuropathic rats (*n* = 6 per group) received intrathecal injection of cynandione A (100 μg) followed by intrathecal normal saline (10 μl) or methyllycaconitine (10 μg) 30 min later. As shown in Figure 6B, postintrathecal injection of methyllycaconitine significantly inhibited cynandione-A-induced mechanical antiallodynia in the ipsilateral hindpaws (*p* < 0.05, by repeated measures two-way ANOVA followed by the post-hoc Student-Newman-Keuls test).

Another four groups of neuropathic rats (*n* = 6 per group) that received the same intrathecal treatments as in the methyllycaconitine prevention study were sacrificed to obtain spinal cords 1 h after the last injection. As exhibited in Figures 6C,D, intrathecal injection of cynandione A remarkably increased mRNA expression of IL-10 and POMC in both contralateral and ipsilateral spinal cords, whereas intrathecal methyllycaconitine was not effective in reducing baseline IL-10 and POMC mRNA expression. However, pretreatment with intrathecal methyllycaconitine entirely blocked cynandione-A-stimulated mRNA expression of IL-10 or POMC (*p* < 0.05, by one-way ANOVA followed by the post-hoc Student-Newman-Keuls test). In addition, intrathecal injection of cynandione A in both contralateral and ipsilateral cords also stimulated the protein expression of IL-10 and β-endorphin, which was entirely attenuated by the pretreatment with intrathecal methyllycaconitine (*p* < 0.05, by one-way ANOVA followed by the post-hoc Student-Newman-Keuls test; Figures 6E,F).

**FIGURE 7:**
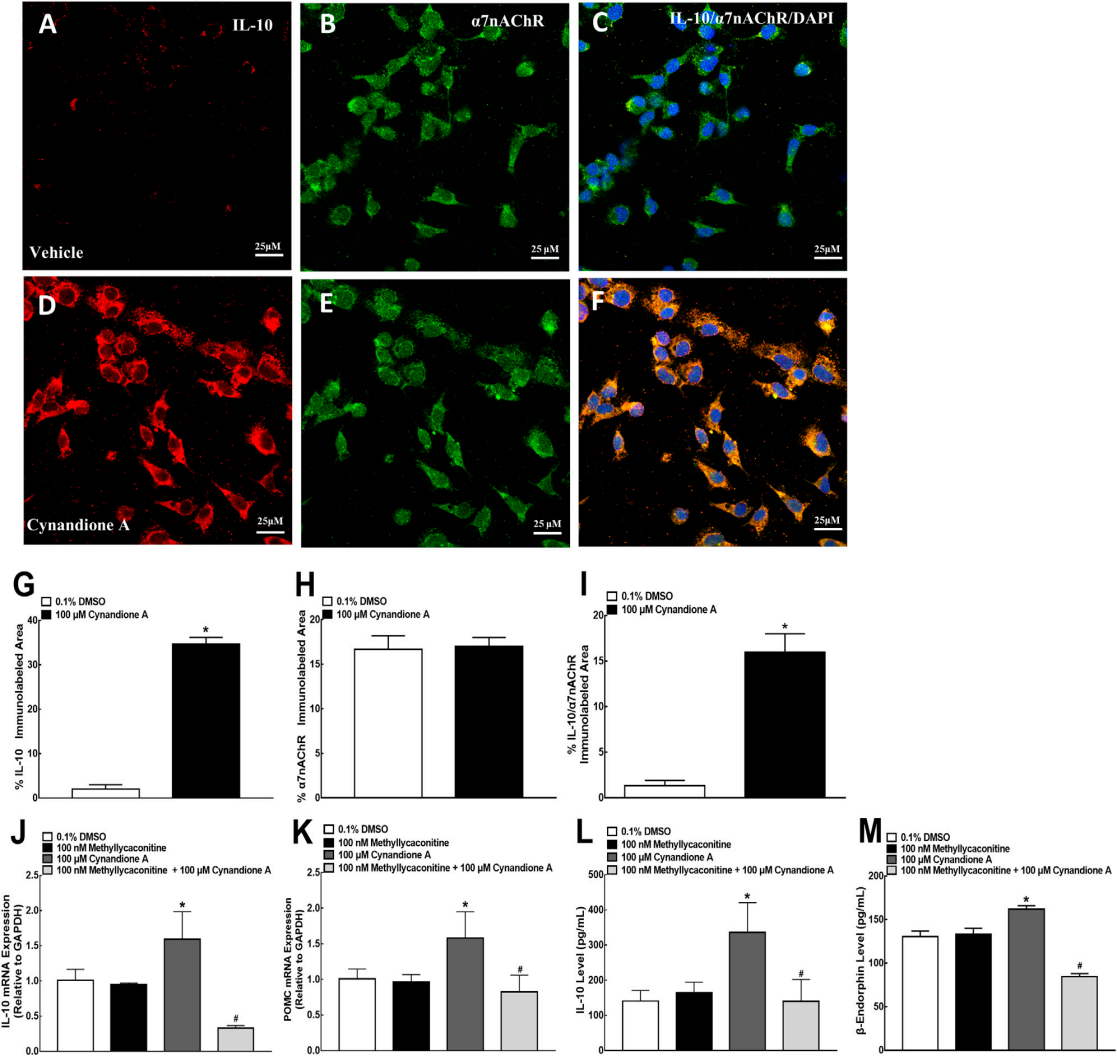
Stimulatory effects of cynandione A on expression of IL-10 colocalized with the α7 nicotinic acetylcholine receptor (α7 nAChR) **(A–I)** and blockade effects of α7 nAChR antagonist methyllycaconitine on cynandione A-stimulated expression of IL-10 and β-endorphin **(J–M)** in cultured microglial cells originated from 1-day-old neonatal rats. Cultured primary microglial cells were collected 2 h after cynandione A incubation. The single and triple immunofluorescence staining of IL-10 (red), α7 nAChR (green), and IL-10 (red)/α7 nAChR (green)/DAPI (blue) was performed. The immunolabeled surface areas of IL-10 (G), α7 nAChR (H), and IL-10/α7 nAChR/DAPI (I) were quantified by using the Image J program. The qRT-PCR and enzyme-linked immunosorbent fluorescent kits were used to detect gene expression and protein secretion in the cells and culture medium, respectively. Data are presented as means ± SEM (*n* = 3 per group with two independent repeats). ^*^ *P* < 0.05 compared with the control group, analyzed by two-tailed and unpaired Student t-test or one-way followed by the post-hoc Student-Newman-Keuls test.

To test cynandione-A-induced expression of IL-10 via α7 nAChR activation directly in microglia, colocalization of IL-10 and α7 nAChRs was assayed in cultured spinal cells. As shown in Figures 7A,C, IL-10 was coexpressed with α7 nAChRs in the vehicle control microglial cells, reflected by single and triple immunostaining under ×60 magnifications. Treatment with cynandione A (100 μM) significantly enhanced expression of IL-10 and IL-10/α7 nAChRs but not α7 nAChRs (Figures 7 D–F). Quantitatively, cynandione A treatment significantly increased immunofluorescence intensity of IL-10 and IL-10/α7 nAChRs/DAPI by 16.3- and 11.0-fold (p< 0.05, by one-way ANOVA followed by the post-hoc Student-Newman-Keuls test), but not α7 nAChRs (Figures 7G–I).

Furthermore, cultured primary microglial cells were pretreated with the vehicle or methyllycaconitine (100 nM, van Goethem et al., 2019) 30 min later followed by cynandione A (100 μM) over 2 h. The cultured cells and culture medium were collected to measure gene and protein expression of IL-10 and β-endorphin. As shown in Figures 7J,K, treatment with cynandione A in cultured microglial cells stimulated mRNA expression of IL-10 and POMC, which was totally blocked by the pretreatment with methyllycaconitine (p< 0.05, by one-way ANOVA followed by the post-hoc Student-Newman-Keuls test), although it did not significantly alter baseline expression of IL-10 or POMC. In addition, cynandione A treatment also stimulated expression of IL-10 and β-endorphin, which was completely inhibited by pretreatment with methyllycaconitine (*p* < 0.05, by one-way ANOVA followed by the post-hoc Student-Newman-Keuls test; Figures 7L,M).

### Cynandione A Stimulated Spinal Microglial Expression of IL-10 and β-Endorphin Through the cAMP/PKA/p38/CREB and IL-10/STAT3 Signaling in an α7-nAChR-Dependent Manner

**FIGURE 8:**
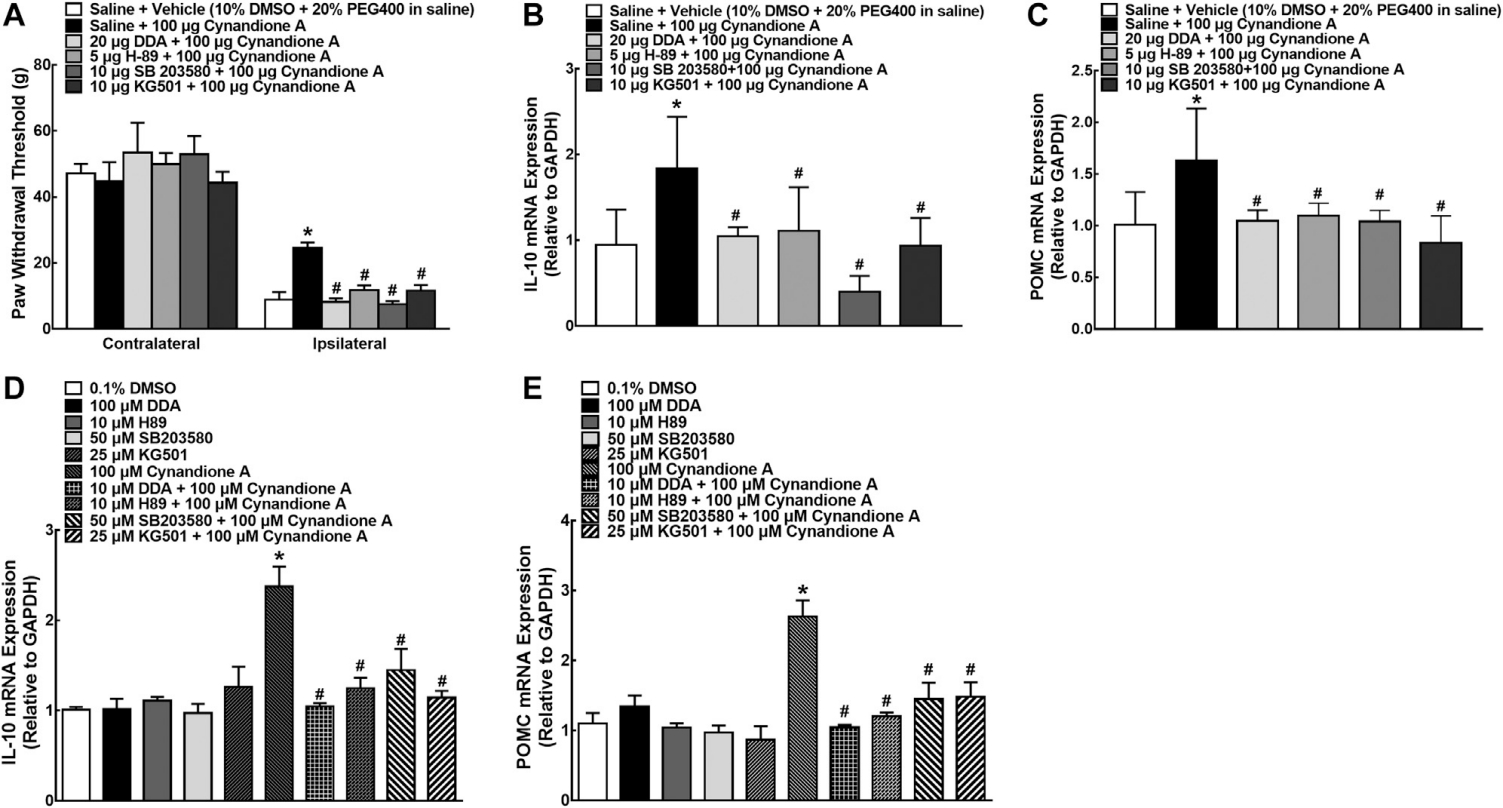
Blockade effects of the adenylyl cyclase inhibitor DDA, PKA activation inhibitor H-89, p38 activation inhibitor SB203580, and CREB activation inhibitor KG501 on cynandione-A-induced spinal mechanical antiallodynia and mRNA expression of IL-10 and the β-endorphin precursor proopiomelanocortin (POMC **(C)**) in the spinal cords of neuropathic rats induced by spinal nerve ligation **(A–C)** and cultured primary microglial cells originated from 1-day-old neonatal rats **(D, E)**. Neuropathic rats received intrathecally the vehicle or DDA, H-89, SB203580 and KG501 30 min followed by cynandione A. The spinal cords and cultured microglial cells were obtained 1 h after the last intrathecal injection or 2 h after incubation to detect IL-10 and POMC gene expression by using qRT-PCR. Data are shown as means ± SEM (*n* = 6 per group in neuropathic rats or *n* = 3 per group with two independent repeats in cultured cells). *, ^#^ *p* < 0.05 compared with the control and cynandione A treatment groups, respectively, analyzed by one-way or two-way ANOVA followed by the post-hoc Student-Newman-Keuls test.

To explore whether the cAMP/PKA/p38/CREB signaling was responsible for cynandione-A-induced mechanical antiallodynia in neuropathic pain and spinal expression of IL-10/β-endorphin, six groups of neuropathic rats (*n* = 6 per group) received intrathecal injection of saline (10 μl) or the specific adenylyl cyclase inhibitor DDA (20 μg, Wu et al., 2017a) PKA activation inhibitor H-89 (5 μg, Wu et al., 2017a), p38 activation inhibitor SB203580 (10 μg Li et al., 2017), and CREB activation inhibitor KG501 (10 μg, Li et al., 2017), respectively, followed by intrathecal injection of cynandione A (100 μg) 30 min later. Intrathecal injection of cynandione A in the ipsilateral hindpaws produced mechanical antiallodynia, which was nearly entirely blocked by the pretreatment with intrathecal DDA, H-89, SB203580, and KG501 (p< 0.05, by repeated measures two-way ANOVA followed by the post-hoc Student-Newman-Keuls test; Figure 8A). Additional six groups of neuropathic rats (*n* = 6 per group) that received the same intrathecal treatments as above were sacrificed 1 h after the last injection. The spinal cords were collected and homogenized to detect gene expression of IL-10 and β-endorphin by using qRT-PCR. As shown in Figures 8B,C, intrathecal injection of cynandione A stimulated spinal mRNA expression of IL-10 and POMC, which was completely reduced by the pretreatment with intrathecal DDA, H-89, SB203580, or KG501 (*p* < 0.05, by one-way ANOVA followed by the post-hoc Student-Newman-Keuls test).

Cultured microglial cells were pretreated with saline or DDA (100 μM, Wu et al., 2017b), H-89 (10 μM, Wu et al., 2017b), SB203580 (50 μM, Li et al., 2017), and KG501 (25 μM, Li et al., 2017) 30 min before cynandione A treatment (100 μM) over 2 h. Treatment with cynandione A in microglial cells stimulated the mRNA expression of IL-10 and POMC, which was significantly inhibited by the pretreatment with DDA, H-89, SB203580, or KG501 (*p* < 0.05, by one-way ANOVA followed by the post-hoc Student-Newman-Keuls test; Figures 8D,E).

**FIGURE 9:**
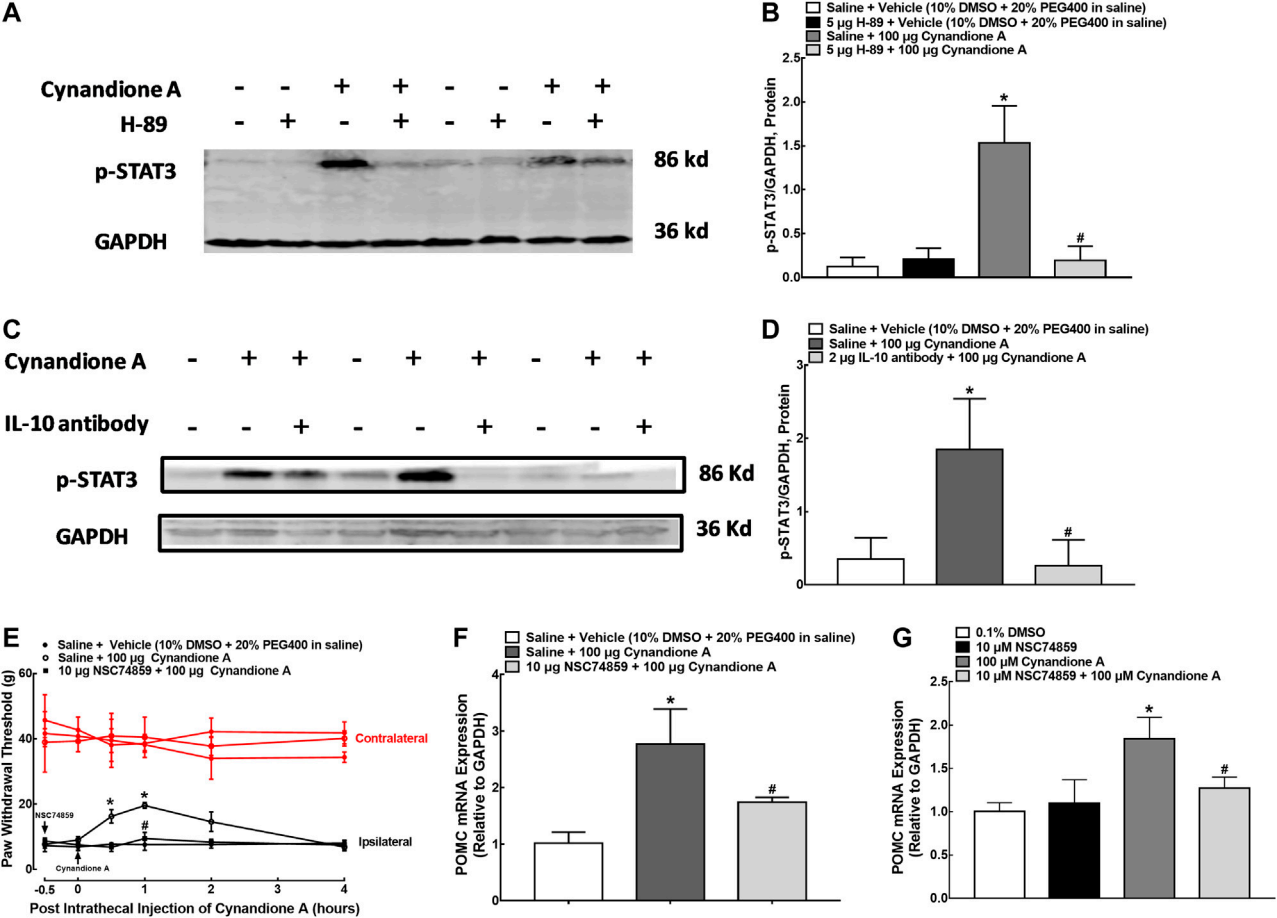
Blockade effects of the PKA activation inhibitor H-89 **(A,B)** and IL-10 antibody **(C,D)** on cynandione-A-stimulated spinal STAT3 phosphorylation in neuropathic rats induced by L5/L6 spinal nerve ligation. Blockade effects of the STAT3 activation inhibitor NSC74859 on cynandione-A-induced spinal mechanical antiallodynia **(E)** and gene expression of the β-endorphin precursor proopiomelanocortin (POMC) in the spinal cords of neuropathic rats **(F)** and cultured primary microglial cells originated from 1-day-old neonatal rats **(G)**. Neuropathic rats received intrathecal H-89, the IL-10 antibody or NSC74859 30 min later followed by intrathecal cynandione A. The spinal cords were obtained 1 h after the last intrathecal injection for detection of STAT3 phosphorylation and POMC expression by using western blot and qRT-PCR, respectively. Data are shown as means ± SEM (*n* = 6 per group in neuropathic rats or *n* = 3 per group with two independent repeats in cultured cells). *, ^#^ *p* < 0.05 compared with the control and cynandione A treatment groups, respectively, analyzed by one-way or measures-repeated two-way ANOVA followed by the post-hoc Student-Newman-Keuls test.

In order to illustrate the role of IL-10/STAT3 signaling in cynandione-A-induced spinal microglial β-endorphin expression and mechanical antiallodynia, the spinal STAT3 phosphorylation was first measured. Four groups of neuropathic rats (*n* = 6 per group) received intrathecal injection of saline (10 μl) or the PKA activation inhibitor H-89 (5 μg) 30 min later followed by the vehicle (10 μl) or cynandione A (100 μg). The rats were sacrificed 1 h after the last injection and the spinal cords were obtained for detection of the STAT3 phosphorylation using western blot. Intrathecal injection of cynandione A stimulated spinal STAT3 phosphorylation. Pretreatment with intrathecal injection of H-89 did not affect baseline phosphorylation of STAT3, but abolished cynandione-A-stimulated STAT3 activation (*p* < 0.05, by one-way ANOVA followed by the post-hoc Student-Newman-Keuls test; Figures 9A,B).

In addition, additional four groups of neuropathic rats (*n* = 6 per group) received intrathecal injection of saline (10 μl) or the IL-10 antibody (2 μg) over 30 min followed by intrathecal injection of the vehicle (10 μl) or cynandione A (100 μg). The rats were sacrificed 1 h after the last injection and the spinal cords were obtained to detect the STAT3 phosphorylation. As shown in Figures 9C,D, pretreatment with the IL-10 antibody totally blocked cynandione-A-stimulated but not baseline STAT3 phosphorylation (*p* < 0.05, by one-way ANOVA followed by the post-hoc Student-Newman-Keuls test). The original gel image in Figure 9A and 9C can be found in the Supplementary Material.

Furthermore, three groups of neuropathic rats (*n* = 6 per group) received intrathecal injection of saline (10 μl) or the specific STAT3 activation inhibitor NSC74859 (10 μg, Wu et al., 2018b) 30 min before intrathecal injection of cynandione A (100 μg). Cynandione A intrathecal injection produced time-dependent mechanical antiallodynia, which was completely inhibited by the pretreatment with intrathecal injection of NSC74859 (*p* < 0.05, by repeated measures two-way ANOVA followed by the post-hoc Student-Newman-Keuls test; Figure 9E). Additional three groups of neuropathic rats (*n* = 6 per group) received the same intrathecal treatments as above. The rats were sacrificed 1 h after the last injection and the spinal cords were obtained. As exhibited in Figure 9F, pretreatment with intrathecal NSC74859 totally blocked cynandione-A-stimulated spinal POMC expression (*p* < 0.05, by one-way ANOVA followed by the post-hoc Student-Newman-Keuls test). In addition, pretreatment (30 min earlier) with NSC74859 (10 μM, Wu et al., 2018b) inhibited 100 μM cynandione-A-stimulated but not baseline expression of POMC in cultured microglial cells (*p* < 0.05, by one-way ANOVA followed by the post-hoc Student-Newman-Keuls test, Figure 9G).

**FIGURE 10:**
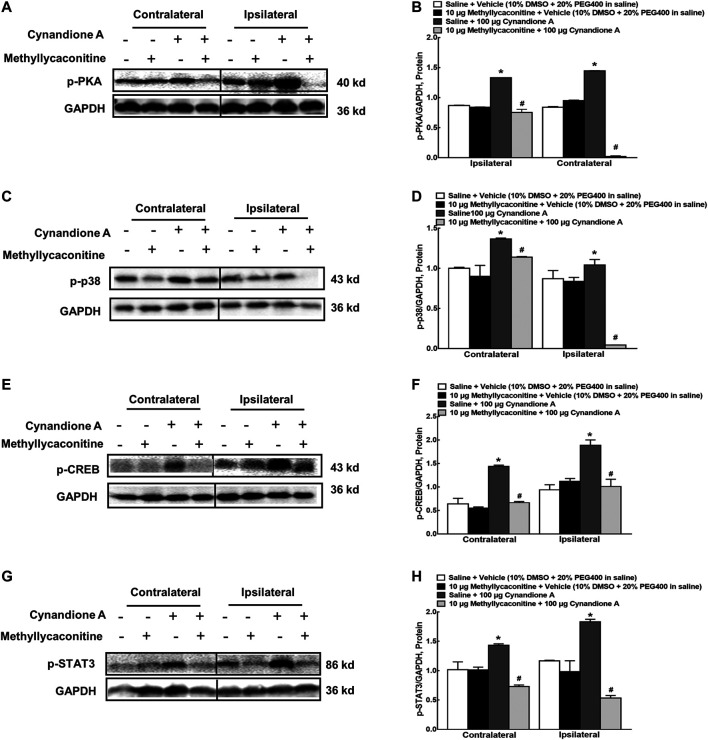
Blockade effects of the α7 nicotinic acetylcholine receptor (α7 nAChR) antagonist methyllycaconitine on cynandione-A-induced phosphorylation of spinal PKA **(A,B)**, p38 **(C,D)**, CREB **(E,F),** and STAT3 **(G,H)** in neuropathic rats induced by L5/L6 spinal nerve ligation. Neuropathic rats received intrathecal injection of the vehicle or methyllycaconitine 30 min later followed by intrathecal cynandione A. The spinal cords were obtained 1 h after the last intrathecal injection for the phosphorylation detection by using western blot. Data are shown as means ± SEM (*n* = 6 per group). *, ^#^ *p* < 0.05 compared with the control and cynandione A treatment groups, respectively, analyzed by one-way ANOVA followed by the post-hoc Student-Newman-Keuls test.

In order to illustrate whether cynandione A activated PKA/p38/CREB/STAT3 signaling following α7 nAChR agonism, four groups of neuropathic rats (*n* = 6 per group) received saline (10 μl) or methyllycaconitine (10 μg) 30 min later followed by intrathecal injection of the vehicle (10 μl) or cynandione A (100 μg). The rats were sacrificed 1 h after the last intrathecal injection and the spinal cords were obtained. Phosphorylation of PKA, p38, CREB, and STAT3 in the contralateral and ipsilateral spinal cords was detected using western blot. As shown in Figures 10A–H, intrathecal injection of cynandione A stimulated phosphorylation of PKA, p38, CREB, and STAT3 in the contralateral and ipsilateral spinal cords. Pretreatment with intrathecal injection of methyllycaconitine entirely blocked cynandione-A-promoted but not baseline PKA, p38, CREB, and STAT3 phosphorylation (*p* < 0.05, by one-way ANOVA followed by the post-hoc Student–Newman–Keuls test). The original gel image in Figure 10A, 10C, 10E and 10G can be found in the Supplementary Material.

## Discussion

Intrathecal injection of cynandione A has been demonstrated to produce mechanical antiallodynia and thermal antihyperalgesia in neuropathic pain through spinal expression of β-endorphin (Huang et al., 2017). Our current study extends that cynandione A induces expression of β-endorphin through spinal microglial expression of IL-10 but not in reverse. The notion is supported by the following facts: (1) intrathecal injection of cynandione A stimulated spinal expression of IL-10 and β-endorphin but not dynorphin A, measured by using qRT-PCR and fluorescent immunoassay kits. Furthermore, intrathecal cynandione A selectively stimulated IL-10 and β-endorphin expression in microglia, but not in astrocytes or neurons, in both contralateral and ipsilateral spinal cords of neuropathic rats, directly identified by double immunofluorescence staining. (2) Pretreatment with intrathecal injection of the IL-10 antibody in neuropathic rats eliminated cynandione-A-induced spinal mechanical antiallodynia and gene expression of POMC but not IL-10. In addition, intrathecal β-endorphin antiserum also attenuated spinal cynandione-A-induced mechanical antiallodynia but not gene expression of POMC or IL-10. On the other hand, intrathecal IL-10 antibody neutralized IL-10 secreted and inhibited expression of β-endorphin, whereas intrathecal β-endorphin antiserum neutralized β-endorphin (but not IL-10) secreted expression. (3) Cynandione A treatment stimulated expression of IL-10 and β-endorphin but not dynorphin A in primary cultures of microglia. (4) Pretreatment with the IL-10 antibody in cultured microglial cells completely inhibited cynandione-A-stimulated gene expression of POMC but not IL-10, whereas the β-endorphin antiserum failed to affect cynandione-A-stimulated gene expression of POMC or IL-10.

It has been reported that α7 nAChRs are colocalized on microglial cells as determined by qRT-PCR, western blot, immunofluorescence, and immunohistochemistry analyses and their activation is associated with antinociception and neuroprotection (Shytle et al., 2004; Zhang et al., 2015; Shi et al., 2016; Zhang et al., 2017). Our current study demonstrated that intrathecal methyllycaconitine, given before or after cynandione A injection, significantly attenuated cynandione-A-induced mechanical antiallodynia in neuropathic rats. In contrast, intrathecal GLP-1 receptor antagonist exendin (9–39), GPR40 antagonist GW1100, and CRF receptor antagonist a-helical CRF (9–41) failed to block cynandione A mechanical antiallodynia (Huang et al., 2017). Additionally, pretreatment with intrathecal methyllycaconitine blocked cynandione-A-stimulated expression of IL-10 and β-endorphin in the spinal cords. More directly, cultured microglial cells coexpressed IL-10 and α7 nAChRs and cynandione A incubation stimulated expression of IL-10 but not α7 nAChRs, which was totally blocked by methyllycaconitine. It was also recently reported that the specific α7 nAChR agonist PHA-543163 stimulated methyllycaconitine-reversible IL-10/β-endorphin expression in cultured microglial cells (Apryani et al., 2020). These results together suggest that cynandione A induces spinal microglial expression of IL-10/β-endorphin and produces antinociception through α7 nAChR activation. However, this conclusion is compromised by lacking elucidated interactions of cynandione A with α7 nAChRs at the molecular level. Particularly, our previous studies also revealed that the spinal microglial IL-10/β-endorphin pathway in bone cancer pain and neuropathic pain mediated the α7 nAChR agonists PHA-543613-, cinobufagin- and lemairamin-induced antinociception, although their chemical structures were different (Apryani et al., 2020; Wang et al., 2020). Further studies are needed to assess the activities of cynandione A, as well as PHA-543613, cinobufagin, and lemairamin, on α7 nAChRs by using radioligand analysis, electrophysiology, or calcium imaging FLIPR assays.

α7 nAChRs generate specific and complex Ca^2+^-dependent signals that include adenylyl cyclase, PKA, protein kinase C, Ca^2+^-calmodulin-dependent kinase (Allen et al., 1996; Chan et al., 2014), and phosphatidylinositol 3-kinase (Kihara et al., 2001; Chan et al., 2014), which trigger cell depolarization and turn on various functional switches (Albuquerque et al., 2009). It was reported that α7 nAChR and G-protein interaction regulated cAMP levels under LPS treatment in microglia (King et al., 2017). In addition, nicotine stimulation led to PKA activation and further Raf-1/MEK/ERK1/2 and JAK2/STAT3 signaling through α7 nAChRs in human oral keratinocyte (Arredondo et al., 2006). Specifically, the serine 365 in the M3-M4 cytoplasmic loop of the α7 nAChR has been demonstrated to be a PKA phosphorylation site (Charpantier et al., 2005), though PKA is customarily activated by G-protein Gαs (Beaulieu and Gainetdinov, 2011). In our current study, cynandione A stimulated PKA phosphorylation in an α7-nAChR-dependent manner, and the adenylate cyclase inhibitor and PKA activation inhibitor blocked cynandione-A-induced spinal microglial expression of IL-10 and subsequent β-endorphin expression. The results suggest that cynandione A stimulates microglial expression of IL-10 following α7 nAChR agonism through the cAMP/PKA signaling.

As a transcription factor, CREB has been shown to be directly phosphorylated at Ser-133 by activated PKA in a classic way or indirectly through several other kinases particularly MAPKs including p38 (Scott Bitner, 2012). It has been demonstrated that both classic Gs-cAMP/PKA/CREB and alternative Gs-cAMP/PKA/p38/CREB mediate GLP-1 receptor agonism-induced expression of M2 microglial biomarkers Arg 1, CD206, IL-4, and IL-10 (Wu et al., 2018a). α7 nAChR agonists biochemically characterized pharmacological induction of CREB phosphorylation which was blocked by the pretreatment with methyllycaconitine (Wan et al., 2016). It has been demonstrated that α7 nAChR activation increased CREB phosphorylation in brain and improved cognitive function (Bitner et al., 2007), but it is not known whether activation of α7 nAChRs stimulates CREB phosphorylation and relieves pain. Our results reveal that cynandione-A-stimulated microglial IL-10/β-endorphin expression is subsequently through CREB phosphorylation via p38 (or likely p38β) activation alternatively. The note is supported by the following findings in our current and previous studies (Huang et al., 2017): (1) intrathecal cynandione A stimulated α7-nAChR-dependent spinal p38 and CREB activation in neuropathic rats; (2) cynandione A upregulated phosphorylation of MAPKs including p38, ERK1/2, and JNK in cultured microglial cells; (3) cynandione-A-stimulated IL-10/β-endorphin expression in microglial cells was completely inhibited by the p38 activation inhibitor SB203580 (but not by the ERK1/2 or JNK activation inhibitors) and CREB activation inhibitor KG501. In addition, knockdown of spinal p38β but not p38α using siRNAs also completely blocked cynandione-A-induced β-endorphin expression; (4) cynandione-A-induced mechanical antiallodynia was totally attenuated by SB203580 and KG501.

It has been demonstrated that the IL-10/JAK/STAT3 pathway in microglia mediates IL-10-stimulated β-endorphin expression, which is in parallel to its inhibition of expression of neuroinflammatory cytokines through anti-inflammatory elements (Wu et al., 2017b; Wu et al., 2018b). We thus postulate that cynandione A stimulates β-endorphin expression through autocrime IL-10 expression via the STAT3 signaling. Activation of α7 nAChRs indeed led to reducing inflammatory drive through a JAK2-STAT3 pathway that couples with CREB/Irs2/Akt survival signaling in the mouse islets (Gupta et al., 2018). α7 nAChR activation increased hypothalamic POMC expression by triggering JAK2/STAT3 pathway (Souza et al., 2019). Consistently, our study demonstrated that cynandione A stimulated microglial STAT3 activation which could be blocked by the α7 nAChR antagonist methyllycaconitine, PKA activation inhibitor H-89, and IL-10 neutralizing antibody. Moreover, the STAT3 activation inhibitor NSC74859 also completely attenuated cynandione-A-induced expression of β-endorphin in the spinal cords of neuropathic rats and in cultured microglial cells. It is noted that ChIP or EMSA experiments are further needed to illustrate cynandione-A-induced interactions between STAT3 and POMC promoter at the molecular level.

In conclusion, cynandione A produces mechanical antiallodynia in neuropathic pain through spinal α7 nAChR activation. The subsequent mechanisms involve microglial IL-10 expression via the cAMP/PKA/p38/CREB signaling and subsequent β-endorphin expression via the autocrime IL-10/STAT3 signaling.

## Data Availability Statement

The datasets presented in this study can be found in online repositories. The names of the repository/repositories and accession number(s) can be found in the article/Supplementary Material.

## Ethics Statement

The animal study was reviewed and approved by the Animal Care and Welfare Committee of Shanghai Jiao Tong University and carried out in accordance with the animal care guidelines of the National Institutes of Health. The consent to participate is not applicable.

## Author Contributions

Q-QH and Y-XW conceived and designed the experiments; Q-QH, MY, Z-YW, HL, and J-PA performed the experiments; Q-QH, MY, and Y-XW analyzed the data; Q-QH and Y-XW prepared the manuscript. All authors read and approved the final manuscript.

## Conflict of Interest

The authors declare that the research was conducted in the absence of any commercial or financial relationships that could be construed as a potential conflict of interest.
