# Supplementary material for: Cynandione A Alleviates Neuropathic Pain Through α7-nAChR-Dependent IL-10/β-Endorphin Signaling Complexes
Source: Front Pharmacol. 2021 Jan 27;11:614450. doi: 10.3389/fphar.2020.614450 (PMC7873367; doi:10.3389/fphar.2020.614450)
Supplement: Supplementary file 2 [file table2.docx]

**Highlights:**

Cynandione A produces antinociception through α7 nAChR activation.

Cynandione A stimulates spinal microglial IL-10 expression and subsequent β-endorphin expression.

Cynandione A induces IL-10 expression via the cAMP/PKA/p38/CREB signaling.

Cynandione A evokes β-endorphin expression via the autocrine IL-10/STAT3 signaling.
