# Supplementary material for: Cynandione A Alleviates Neuropathic Pain Through α7-nAChR-Dependent IL-10/β-Endorphin Signaling Complexes
Source: Front Pharmacol. 2021 Jan 27;11:614450. doi: 10.3389/fphar.2020.614450 (PMC7873367; doi:10.3389/fphar.2020.614450)
Supplement: Supplementary file 4 [file table4.docx]

**Replies to editors (in red)**

Two reviewers have submitted their evaluations of your manuscript. I should agree with them that while we were all impressed with an abundance of time and energy you invested in generating and presenting a large amount of data in totally 13 figures (not including a schematic illustration of working mechanism in Fig 14), the story looks rather incohesive, disorganized and superficial, drafting away from the key point of the title, the role of AcHRa7 in CynA inhibition of neuropathic rats, which is potentially interesting. In this regard, the title should be streamlined and changed to” Cynandione A alleviates neuropathic pain through activation of nicotinic acetylcholine receptor a7 subunit, or in an nACha7R dependent mechanism”. Overall, this manuscript really needs a substantial revision, reorganization and more experimental evidence to make a compelling story to publish in Frontiers in Neuroscience.

We appreciate the editor’s comments and have performed additional studies and substantially revised the manuscript (including reducing figures to 10) to address our major discovery. Our previous paper demonstrated that cynandione A stimulated β-endorphin expression (Huang et al., Brain Behavior Immun., 2017). This paper extends to explore its upstream signaling and target molecule mechanisms, and concludes that cynandione A produces antineuropathic pain through spinal microglial expression of IL-10 and subsequent expression of β-endorphin following α7nAChR activation. To avoid the partial overlap with our previous publication, the title of the current paper has been changed to “Cynandione A alleviates neuropathic pain in an α7nAChR-dependent mechanism” as suggested.

Major concerns

1. The likely novel discovery of this study is the interrelationship between CynA and nACha7 in neuropathic pain. Unfortunately, only two (Fig. 7 and 13) out of 13 figures dealt with this issue. The data from the rest figures seem rather irrelevant or loosely related to the main discovery.
2. The evidence for CynA and nAChRa7 interaction looks very thin, largely dependent on single antagonist. Based on this, it is even not proper to make a title as “CynA ….. by activation of nACha7”.
3. Can CynA modulate nAChRa7 binding and gating properties? This is critical data needed to make the current title even legitimately.

We appreciate and fully understand the editor’s comments. In this paper we extended to explore the upstream signaling of β-endorphin and its target molecule, and hypothesized that cynandione A produced antineuropathic pain through spinal microglial expression of IL-10 and subsequent expression of β-endorphin in an α7nAChR-depdent mechanism. In the regard of α7nAChRs, we have demonstrated that 1) intrathecal the α7nAChR antagonist methyllycaconitine blocked cynandione A-induced mechanical antiallodynia and stimulation of IL-10/β-endorphin expression in the spinal cords of neuropathic rats; 2) Cultured microglial cells coexpressed IL-10 and α7 nAChRs and cynandione A treatment stimulated expression of IL-10 (but not α7 nAChRs), which was totally blocked by methyllycaconitine; 3) Methyllycaconitine treatment also attenuated cynandione A-stimulated PKA, p-38, CREB and STAT3 phosphorylation in primary microglial cells. However, we did not provide α7nAChR binding or gating data due to not access the techniques in house and unsuccessful collaborative efforts with a research institute. To acknowledge the limits of the study, the title of the paper has been changed to “Cynandione A alleviates neuropathic pain in an α7 nAChR-dependent mechanism” as suggested, and a paragraph has been added in the Discussion section: “However, this conclusion is compromised by lacking elucidated interactions of cynandione A with α7 nAChRs at the molecular level. Further studies are needed to assess cynandione A efficiency on α7 nAChRs expressed in the Xenopus oocytes or other cell lines, by using radioligand analysis, electrophysiology or calcium imaging FLIPR assay”.

4. Can intrathecal nACha7 antagonist block the inhibition of pain hypersensitivity by systemic administration CynA?

We appreciate and fully understand the reviewer’s points. We did not conduct the suggested experiment as we did not have sufficient amount of cynandione A to offer systemic administration. The compound is obtained by extraction and purification and is not commercially available. However, we believe that intrathecal α7nAChR antagonist can effectively block systemic cynandione A-induced antinociception as the spinal cord appears to be the major action site.

5. Does nAChRa7 antagonist reverse CynA pain inhibition if applied after CynA?

As requested, we have performed additional study in which the α7nAChR antagonist methyllycaconitine applied after cynandione A also effectively inhibited cynandione A antinociception. The results are added in the newly revised Fig. 6B. Thank you.

6. Can CynA alter nACha7 expression level?

We appreciate the reviewer’s suggestion and have conducted a new study in which cynandione A incubation in cultured spinal microglial cells for 2 hours did not alter α7 nAChR expression. The data has added in the newly revised Fig. 7A-7I.

7. There are redundant results (in Fig. 6 and Fig. 10) that were published previously. These two figures need to be removed from this manuscript.

We are sorry for not being able to make Figs. 6 and 10 clearer. These two figures are somewhat similar to the previously published ones but provide more important data that cynandione A induced the expression of IL-10 and subsequent expression of β-endorphin. As suggested, these two figures have been changed/integrated to Figs. 5 and 8.

8. What is the evidence for schematic illustration of a working mechanism presented in Fig. 14?

Is there any prove for this sequential order of signaling pathways activated by CynA? For instance, Inhibition of IL-10 prevented CynA-induced State3 upregulation as shown in Fig. 12 C but not upstream targets such as PKA and p38. There is a possibility that CynA activates all signals directly.

2. The authors showed that nAChRa7 antagonist reduced CynA-induced phosphorylation of 4 different signaling pathways in Fig. 13, it is unclear why the authors had to test so many pathways.

We appreciate and fully understand the reviewer’s concerns. It is known that IL-10 produces biological functions such as β-endorphin expression via the STAT3 phosphorylation. Meanwhile activation of α7 nAChR, like GLP-1 receptors, has been reported to stimulate IL-10 expression through the upstream signals which include cAMP/PKA/p38/CREB pathway ([Allen et al., 1996](#_ENREF_3); [Chan et al., 2014](#_ENREF_14); Wu et al., 2017a,b; Wu et al., 2018a,b). Thus the IL-10 antibody would block cynandione A-induced activation of STAT3 but not its upstream signals such as PKA and p38. The related discussion has been modified to better illustrate the signaling pathways.

Minor comments

1. Fig. 7A should separate the data of contralateral and ipsilateral CynA injections so that the curves of nAChRa7 antagonist can be fully separable.

We appreciate the reviewer’s suggestion and have deleted the contralateral data to make the lines of methyllycaconitine separable. Thank you.

3. The quality of the images in Fig. 13 E and G are very poor. The authors should give the full gel images of this figure.

We appreciate the reviewer’s concerns and have replaced gel imagines in Fig. 13E and G (now Fig. 10E and 10G in the revised version).

5. 342, change to “4-hour observation”

6. 358, please avoid using “To confirm….”(not sounds scientific), change to “To test ….”

7. Also 358, change to “the specific expression of spinal microglial IL-10..”

8. 369, change to “ As revealed by a confocal microscope with x30 magnification, …”

9. 386, change to “ in cultured spinal microglia”.

10. 642, change to “Our current study further extends that..”.

11. 648, change to “selectively…”

All suggested changes have been made in the revised manuscript, Thank you.

4. The introduction is too long and not focusing.

12. Both Introduction and Discussion are too long and not focusing on the main and navel findings, nACHRa7 or IL-10?? Especially for Discussion, it should be reorganized and shorten to half of the current length.

We appreciate your suggestion and have substantially edited the manuscript and made it significantly shorter.

The interactive review of your manuscript "Title page Cynandione A alleviates neuropathic pain through spinal microglial interleukin-10/β-endorphin expression following α7 nicotinic acetylcholine receptor activation" submitted to Frontiers in Pharmacology, section Neuropharmacology has now been activated.

The reviewers recommended that you make substantial amendments to your manuscript. Please respond within the next 21 days to all comments raised by the reviewers and editor in the online review forum. You can also submit a revised version of your manuscript at that time. We encourage you to submit your documents with tracked changes to highlight the revisions.

There can be more than one iteration between authors and reviewers, but only when all comments by each reviewer have been addressed successfully can the review be finalized.

To access the review forum and respond to the reviewers, please click on the following link:
<http://www.frontiersin.org/Review/EnterReviewForum.aspx?activationno=7ca71c10-541f-42f6-91a8-45adc37e5274>
