# Supplementary figures and images for: Cynandione A Alleviates Neuropathic Pain Through α7-nAChR-Dependent IL-10/β-Endorphin Signaling Complexes
Source: Front Pharmacol. 2021 Jan 27;11:614450. doi: 10.3389/fphar.2020.614450 (PMC7873367; doi:10.3389/fphar.2020.614450)

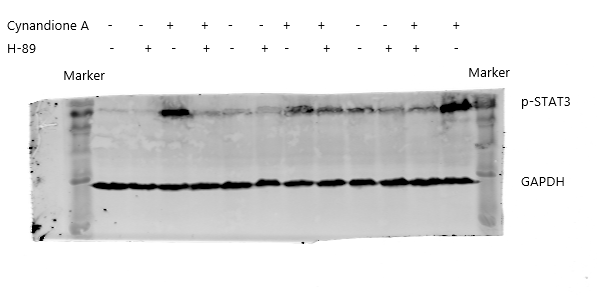

Supplement: Supplementary file 5 [file image1.tiff]

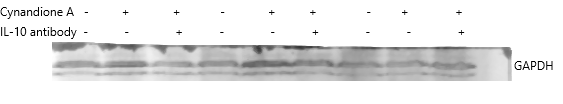

Supplement: Supplementary file 6 [file image2.tiff]

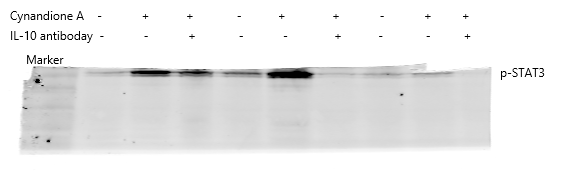

Supplement: Supplementary file 7 [file image3.tiff]

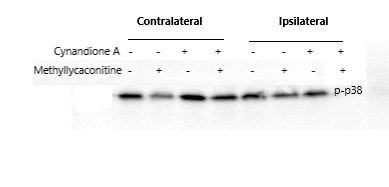

Supplement: Supplementary file 8 [file image4.tiff]

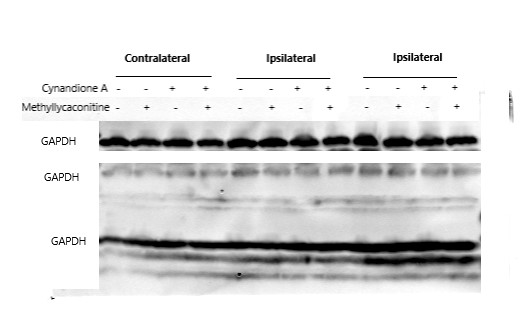

Supplement: Supplementary file 9 [file image5.tiff]

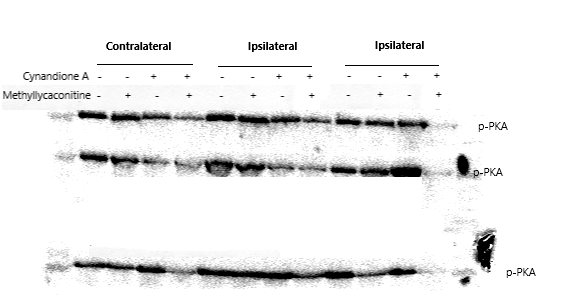

Supplement: Supplementary file 10 [file image6.tiff]

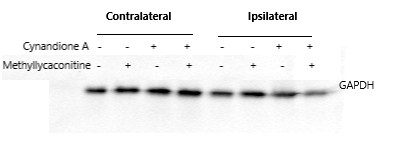

Supplement: Supplementary file 11 [file image7.tiff]

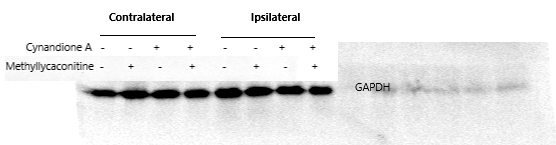

Supplement: Supplementary file 12 [file image8.tiff]

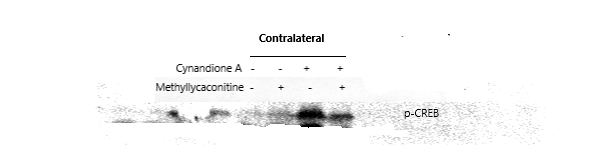

Supplement: Supplementary file 13 [file image9.tiff]

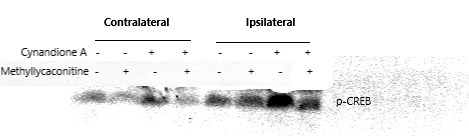

Supplement: Supplementary file 14 [file image10.tiff]

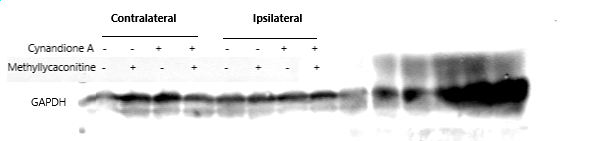

Supplement: Supplementary file 15 [file image11.tiff]

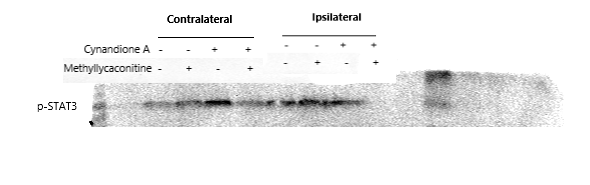

Supplement: Supplementary file 16 [file image12.tiff]

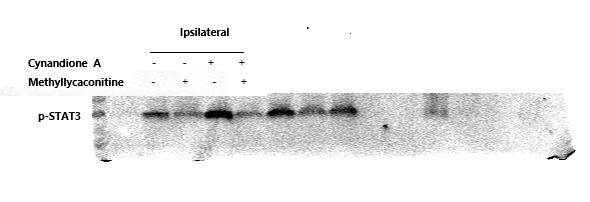

Supplement: Supplementary file 17 [file image13.tiff]

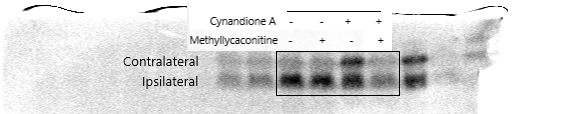

Supplement: Supplementary file 18 [file image14.tiff]

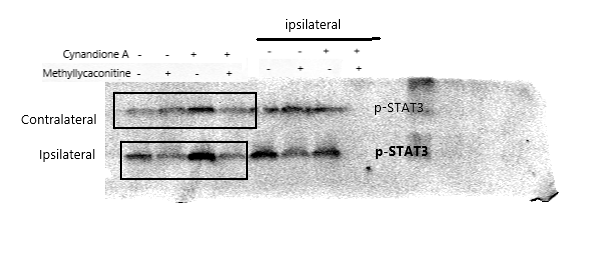

Supplement: Supplementary file 19 [file image15.tiff]
