# Supplementary material for: Cynandione A Alleviates Neuropathic Pain Through α7-nAChR-Dependent IL-10/β-Endorphin Signaling Complexes
Source: Front Pharmacol. 2021 Jan 27;11:614450. doi: 10.3389/fphar.2020.614450 (PMC7873367; doi:10.3389/fphar.2020.614450)
Supplement: Supplementary file 1 [file table1.docx]

### October 6, 2020

#### Dear Editor,

We would like to submit the manuscript titled “Cynandione A alleviates neuropathic pain through spinal microglial expression of IL-10/β-endorphin following α7 nAchR activation” as an original article for publication in *Frontiers in Pharmacology.*

*.*

Cynandione A, an acetophenone isolated from Cynanchum Wilfordii Radix, exhibits antihypersensitivity effects in neuropathic pain. This study sought to explore the target molecule and mechanisms underlying cynandione A mechanical antiallodynia in neuropathic pain. Intrathecal injection of cynandione A significantly attenuated mechanical allodynia in neuropathic rats and substantially increased spinal expression of IL-10 and β-endorphin but not dynorphin A. Cynandione A treatment also increased IL-10 and β-endorphin expression in cultured primary microglia. The IL-10 antibody attenuated cynandione A-induced spinal or microglial gene expression of β-endorphin and mechanical allodynia, whereas the β-endorphin antiserum blocked cynandione A-induced mechanical allodynia but not spinal or microglial IL-10 gene expression. The α7 nAchR antagonist methyllycaconitine significantly declined cynandione A-induced mechanical antiallodynia and spinal or microglial expression of IL-10 and β-endorphin. Cynandione A stimulated microglial phosphorylation of PKA, p38 and CREB, which was inhibited by methyllycaconitine. Treatment with the adenylyl cyclase inhibitor DDA, PKA inhibitor H-89, p38 inhibitor SB203580 and CREB inhibitor KG501 attenuated cynandione A-induced mechanical antiallodynia and spinal or microglial expression of IL-10 and β-endorphin. Cynandione A stimulated spinal phosphorylation of the transcription factor STAT3, which was inhibited by methyllycaconitine, H-89 and the IL-10 antibody. The STAT3 inhibitor NSC74859 weakened cynandione A-induced mechanical antiallodynia and spinal expression of β-endorphin.

We believe that our findings are of great interest to the readers of *Frontiers in Pharmacology*, since our results illustrate that cynandione A produces mechanical antiallodynia through spinal microglial expression of IL-10 via the cAMP/PKA/p38/CREB signaling and subsequent β-endorphin expression via the IL-10/STAT3 signaling, following α7 nAchR activation.

With the submission of this manuscript, we would like to undertake that 1) the above mentioned manuscript has not been published, accepted for publication or under editorial review for publication elsewhere; 2) the authors have read and approved to submit it to *Frontiers in Pharmacology*; 3) the authors have declared that no competing interests exist.

Thank you for your consideration.

Sincerely,

Yong X. Wang, MD & PhD

Professor
King's Lab
Shanghai Jiao Tong University School of Pharmacy
Shanghai 200240, China

Tel./Fax: 86-21-3420-4766
Email: [yxwang@sjtu.edu.cn](mailto:yxwang@sjtu.edu.cn)
