# Supplementary material for: Cynandione A Alleviates Neuropathic Pain Through α7-nAChR-Dependent IL-10/β-Endorphin Signaling Complexes
Source: Front Pharmacol. 2021 Jan 27;11:614450. doi: 10.3389/fphar.2020.614450 (PMC7873367; doi:10.3389/fphar.2020.614450)
Supplement: Supplementary file 3 [file table3.docx]

Dear editor：

These are the supplementary materials for the cropped images of blots are shown in figures, as shown in the supplementary materials we submitted, most of them are not the entire original gel(s), because when we do the western blot experiment, we would like to cut the gel before transferring the proteins to PVDF membrane, so that we get a full scan of the membrane but not the entire original gel(s). And for some gel such as 12 A, the p-STAT3 and GAPDH were transferred together on the same PVDF membrane, but for other gels like 12 B and 13A\C\E\G, the proteins were separated and transferred on the different PVDF membrane. Meanwhile, some gels like 13A, the proteins came from the different gels such as p-PKA or GAPDH were transferred on the same or different PVDF membrane but scan at the same time so the same protein showed in the same image. For 13 E and 13 G, the same samples were run in the different gel twice, so we use the same control images.

Wish our supplementary materials can meet the requirements and standards of your journal.

Thank you.

Sincerely,

Yong X. Wang, MD & PhD

Professor
King's Lab
Shanghai Jiao Tong University School of Pharmacy
Shanghai 200240, China

Tel./Fax: 86-21-3420-4766
Email: yxwang@sjtu.edu.cn
